# Design and control of atmospheric water electrolysis systems for green hydrogen in arid environments

**DOI:** 10.1038/s41598-026-68866-z

**Published:** 2026-09-09

**Authors:** Ghada A. Elgamal, Adel B. El-Shabasy, Mostafa Abdelkhalek, Ashraf M. Hamed, Adham Mohamed Abdelkader

**Affiliations:** 1 Mechatronics Engineering Department, Faculty of Engineering and Technology, Egyptian Chinese University, Cairo, Egypt; 2 Design and Production Engineering Department, Faculty of Engineering, Ain Shams University, Cairo, Egypt; 3 Mechanical Power Engineering Department, Faculty of Engineering, Ain Shams University, Cairo, Egypt; 4 Automotive Engineering Department, Faculty of Engineering, Ain Shams University, Cairo, Egypt

**Keywords:** Green hydrogen, Direct air electrolysis, Atmospheric moisture harvesting, Alkaline electrolyzer, ANSYS simulation, PID regulation, Levelized cost of hydrogen, Vapour-compression dehumidifier, Energy science and technology, Engineering, Environmental sciences

## Abstract

Freshwater scarcity limits green hydrogen production in arid regions like the Middle East and North Africa (MENA), despite high solar potential. This study compares two atmospheric-moisture-based systems: a Direct Air Electrolyzer (DAE) using a hygroscopic H₂SO₄ electrolyte in a porous sponge, and a hybrid dehumidifier–alkaline electrolyzer (DH-AE) that condenses ambient moisture via vapor-compression refrigeration. Both are designed for Cairo’s worst-case relative humidity (RH = 38.19%). The four-module DAE sizing is anchored to a platinum-referenced electrochemical ceiling (1.686 V per module, 87.8% energy efficiency, 97.3% electrolysis efficiency). For the SS‑904 L stainless-steel prototype employed herein, an electrode-material correction (80–140 mV penalty) yields a realistic operating band of 1.766–1.826 V and an energy efficiency of 81.1–83.9%, which brackets the measured prototype range of 1.82–1.95 V. The DH‑AE achieves equivalent output at 68.5% overall efficiency. Thermal simulations (ANSYS) confirm safe operation of SS‑904 L electrodes within the iso-corrosion envelope. A closed-loop control framework (humidity/temperature sensing, PID current regulation, SCADA) is proposed and validated in simulation, enabling autonomous operation. Techno-economic analysis for 50–1,000 kg·day⁻¹ shows a benchmark levelized cost of hydrogen (LCOH) of 4.7 USD·kg⁻¹ for the DAE at 1,000 kg·day⁻¹. When full maintenance, electrolyte replenishment, and long-term degradation are included, the fully loaded LCOH rises to approximately 5.8 USD·kg⁻¹—yet this remains roughly 15% lower than the comparable DH‑AE estimate. Prototype implementations are presented. Overall, atmospheric-moisture-based electrolysis offers a viable, decentralized, water-independent pathway for green hydrogen in arid environments.

## Introduction

The global transition toward net-zero energy systems has established hydrogen as a critical energy carrier for sectors that are difficult to electrify directly, including heavy transportation, long-duration energy storage, and carbon-intensive industries such as steel, ammonia, and methanol production^1–4^. Among the available hydrogen-production pathways, water electrolysis powered by renewable electricity—commonly referred to as green hydrogen—offers a carbon-free production route compatible with the rapidly declining costs of solar photovoltaic (PV) and wind energy technologies^5,6^. The role of this green hydrogen is particularly prominent in the automotive sector, where hydrogen fuel cell vehicles represent a key application that enhances the economic viability of hydrogen produced via electrolysis^7^. Nevertheless, conventional water electrolysis requires approximately 9 kg of high-purity water per kilogram of hydrogen produced^8^, creating a critical water–energy conflict in regions possessing the highest solar-energy potential, including the Middle East and North Africa (MENA), Sub-Saharan Africa, the southwestern United States, and Central Asia, where freshwater scarcity is severe^9,10^.

Recent advances in artificial intelligence have significantly improved water treatment and purification technologies, enabling intelligent resource management and supporting emerging approaches for sustainable hydrogen production from alternative water sources, such as atmospheric moisture harvesting^11^.Seawater electrolysis and desalination-assisted electrolysis have been proposed as partial mitigation strategies; however, both approaches introduce substantial materials-compatibility challenges, including chloride evolution and membrane fouling, while also imposing significant capital and energy penalties associated with desalination pre-treatment^12–14^. An emerging and potentially transformative alternative involves extracting the required feedwater directly from atmospheric moisture, thereby fundamentally decoupling hydrogen production from conventional liquid-water supply infrastructure. Two complementary technological architectures have emerged within this rapidly developing research field. The first is the Direct Air Electrolyzer (DAE), originally demonstrated by Guo et al.^15^ and subsequently extended through deliquescent-salt and radiative-cooling approaches^16,17^. In this configuration, a hygroscopic electrolyte (e.g., H₂SO₄, potassium acetate, or NaClO₄) absorbs atmospheric water vapour and enables in-situ electrolysis at the liquid–vapour interface without the need for external liquid-water supply. The second architecture is the hybrid dehumidifier–alkaline electrolyzer (DH-AE) system, in which a refrigeration-based dehumidification unit first condenses atmospheric moisture into liquid water, which is subsequently supplied to a conventional alkaline electrolyzer.

The hygroscopic materials that enable this class of device belong to a rapidly broadening technology domain whose applications extend well beyond hydrogen production, and the maturity of that domain is directly relevant to the prospects of atmospheric water electrolysis. Sorption-based atmospheric water harvesting has advanced from proof-of-concept demonstrations to field-deployed systems capable of extracting potable water at relative humidities below 20%, driven by the development of metal–organic frameworks, salt-in-hydrogel composites, and hierarchically structured polymeric sorbents with tunable water-uptake isotherms^18,19^. Related work has extended moisture harvesting to marine and coastal boundary-layer environments, where persistently elevated humidity above the ocean surface offers a favourable but corrosively demanding operating regime^20^. In agriculture, hygroscopic composites have been integrated into soil-conditioning and passive irrigation systems that capture night time moisture and release it during the day time, reducing external irrigation demand in water-stressed cultivation^21^. A parallel body of work has combined hygroscopic sorbents with radiative cooling and solar-thermal regeneration to achieve passive or near-passive operation without electrical input^17,22^.

These developments matter to the present study in two respects. First, they establish that the sorbent-side limitation identified in Sect. 6.2.1, namely the modest gravimetric uptake of the acid-saturated glass-foam matrix employed here, is addressable with materials that already exist rather than requiring fundamental discovery. Second, they indicate that the balance-of-plant challenges of atmospheric moisture capture, including dust management, condensate handling, and cyclic regeneration, are being solved in adjacent application domains from which hydrogen-production systems can draw directly. The atmospheric water electrolysis concept examined in this work is therefore best understood not as an isolated device concept but as one branch of a maturing hygroscopic-materials technology base.

Emerging developments in electrolysis modelling and atmospheric-resource harvesting have highlighted the importance of multi-physics and system-level approaches for improving performance prediction, scaling behaviour, and integration with renewable-energy systems, particularly in PEM and hybrid electrolyzer configurations^23^. Integrated studies of hydrogen production and storage under realistic operating conditions have further demonstrated that coupling electrolysis with storage and supervisory system modelling can substantially improve both operational predictability and economic feasibility under variable environmental conditions^24^. In parallel, electrically driven surface processes for direct air resource utilization—such as the reversible surface mineralization of atmospheric CO₂ for direct air capture demonstrated by Liu et al.^25^—further emphasize the growing relevance of ambient-environment-responsive electrochemical systems. Moreover, the broader hydrogen value chain—including downstream end-use applications—underscores the growing importance of adaptive and environment-responsive control strategies^26,27^.

Despite growing interest in atmospheric-moisture-based hydrogen production, several significant research gaps remain unresolved. First, no rigorous side-by-side thermodynamic and techno-economic comparison of DAE and DH-AE architectures operating under identical climatic boundary conditions has been reported in the open literature. Second, reproducible climate-informed sizing methodologies grounded in worst-case environmental design conditions remain largely absent from the literature. Third, control strategies capable of addressing the coupled mass-transfer and electrochemical dynamics unique to atmospheric-water-based systems have not yet been comprehensively developed; conventional open-loop control is insufficient under highly variable humidity conditions and fluctuating PV power input^28^.

Beyond hydrogen generation, hygroscopic and moisture-sorbent materials are enabling a broad family of atmospheric-resource technologies, including all-weather atmospheric water harvesting, moisture capture from the ocean surface for off-grid freshwater supply, and passive humidity regulation for smart-farming and controlled-environment agriculture^23,24^. Recent advances have further demonstrated the versatility of hygroscopic materials in emerging applications such as ocean-surface moisture harvesting for off-grid freshwater production and smart farming systems with passive humidity regulation. Notably, studies published in *Advanced Fiber Materials*^29^, *Advanced Materials*^30^, and *Nature Reviews Materials*^31^ highlight the rapid expansion of atmospheric water harvesting technologies across multiple domains. These developments reinforce the relevance of integrating hygroscopic material science with system-level hydrogen production approaches, as pursued in the present work.

The present work addresses these gaps through four principal contributions: (i) development of a first-principles sizing methodology for a four-module DAE stack producing 0.5 L·h⁻¹ of hydrogen under the worst-case monthly minimum relative humidity conditions of Cairo, Egypt (RH = 38.19%, MERRA-2, 2021–2022); (ii) comparative sizing of a DH-AE system using compact heat-exchanger and refrigeration-cycle analysis to achieve equivalent water throughput; (iii) development of a mechatronic control framework integrating humidity, temperature, flow, and pressure sensing with PID-regulated actuation and SCADA-based supervisory control; and (iv) a comparative techno-economic assessment mapping the operational capacity–humidity domains in which each architecture is preferable.

The remainder of this paper is organized as follows. Section 2 presents the climatic boundary conditions and system architectures. Section 3 details the thermodynamic and sizing methodologies. Section 4 describes the ANSYS thermal analysis, while Sect. 5 presents the proposed mechatronic control framework. Sections 6 and 7 discuss the system performance and techno-economic analyses, respectively. Prototype implementation, practical limitations, and concluding remarks are presented in Sects. 8–10.

## Climate data and system architectures

### Climatic boundary conditions

Both architectures were sized using worst-case climatic conditions for Cairo, Egypt (30.06°N, 31.22°E), obtained from the NASA POWER MERRA-2 reanalysis dataset^32^. Monthly mean values of 2-m relative humidity (RH2M), 2-m air temperature (T2M), specific humidity (QV2M), and 10-m wind speed (WS10M) were retrieved for the 2021–2022 period. The principal design parameter was the minimum monthly mean relative humidity recorded over the study period. For Cairo, this value corresponds to RH = 38.19%, determined as the arithmetic mean of the May 2021 minimum (36.69%) and the May 2022 minimum (39.69%), and was adopted as the binding constraint for all subsequent sizing calculations. Table 1 summarizes the complete climatic dataset.

**Table 1:** NASA POWER MERRA-2 climate data for Cairo, Egypt (2021–2022). The worst-case design condition RH = 38.19% (May average of both years) constitutes the binding sizing constraint for all calculations.

Parameter	Year	Jan	Feb	Mar	Apr	May	Jun	Jul	Aug	Sep	Oct	Nov	Dec	Ann.
T2M (°C)	2021	14.74	14.27	14.82	17.45	22.81	24.25	27.36	27.96	26.06	22.96	20.24	15.02	20.70
T2M (°C)	2022	12.09	12.93	13.08	17.58	21.10	25.31	26.77	27.69	26.49	23.11	19.50	17.37	20.29
QV2M (g/kg)	2021	7.39	7.02	7.26	7.69	9.89	12.02	14.28	15.01	13.24	11.17	9.95	7.20	10.19
QV2M (g/kg)	2022	6.23	6.65	6.65	8.54	9.64	12.63	13.67	14.59	13.67	11.17	8.73	8.54	10.07
RH2M (%)	2021	70.94	70.88	70.38	65.12	61.06	66.06	65.25	66.31	64.75	65.69	68.31	68.81	66.94
RH2M (%)	2022	71.75	73.44	71.81	71.12	65.88	65.75	64.62	64.81	65.12	64.31	62.81	70.50	67.62
WS10M (m/s)	2021	4.94	4.81	5.17	4.85	3.95	4.45	4.98	4.90	5.16	4.20	4.14	4.92	4.70
WS10M (m/s)	2022	5.66	5.04	5.31	4.47	4.40	4.64	5.31	4.47	4.52	4.72	4.03	4.14	4.73

**Fig. 1:**
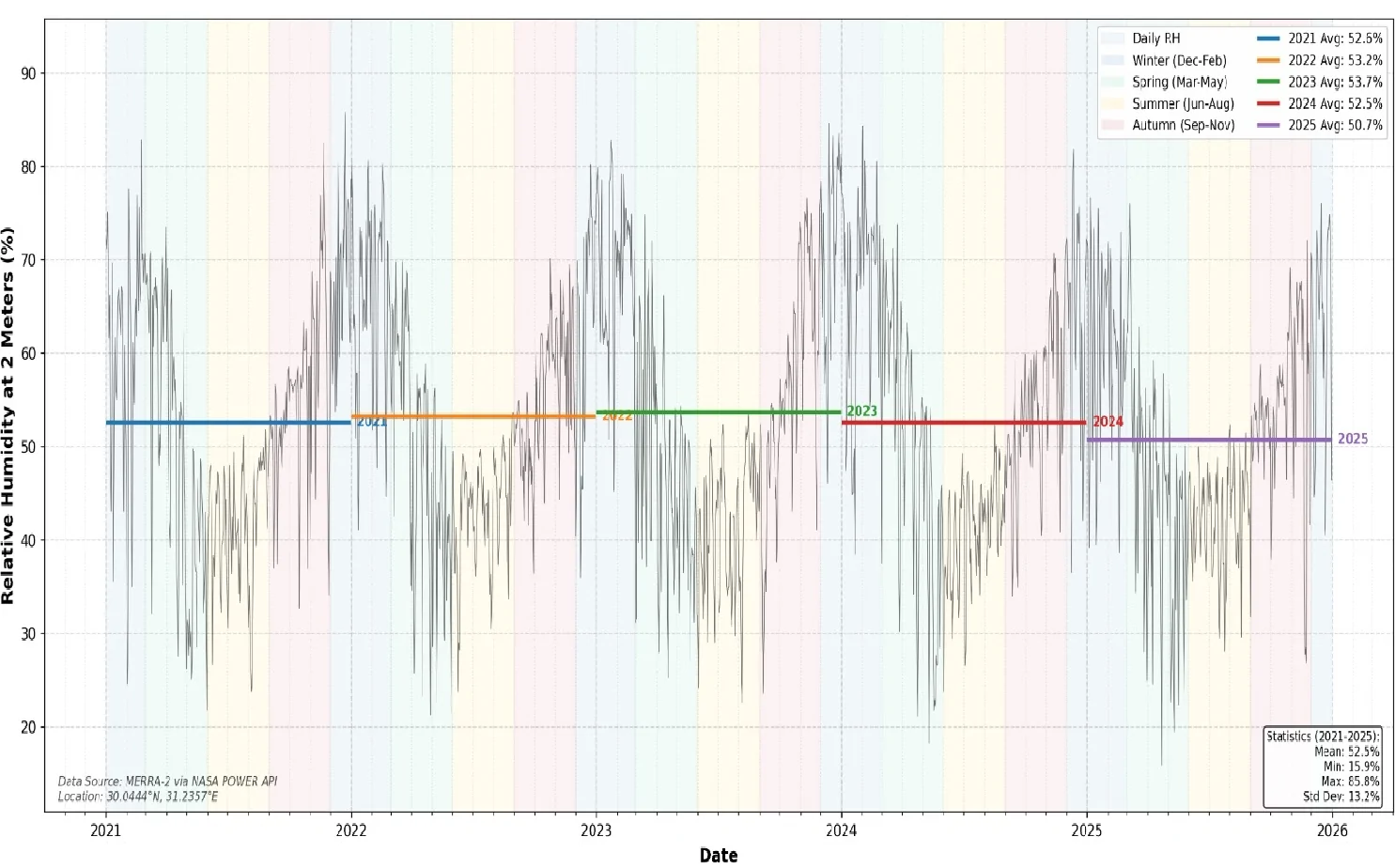
Monthly mean 2 m relative humidity at Cairo (2021–2025) derived from NASA POWER MERRA-2 data. Seasonal variation is evident, with the annual minimum (~ 38–39%) consistently occurring in May–June and forming the worst-case design basis.

**Fig. 2:**
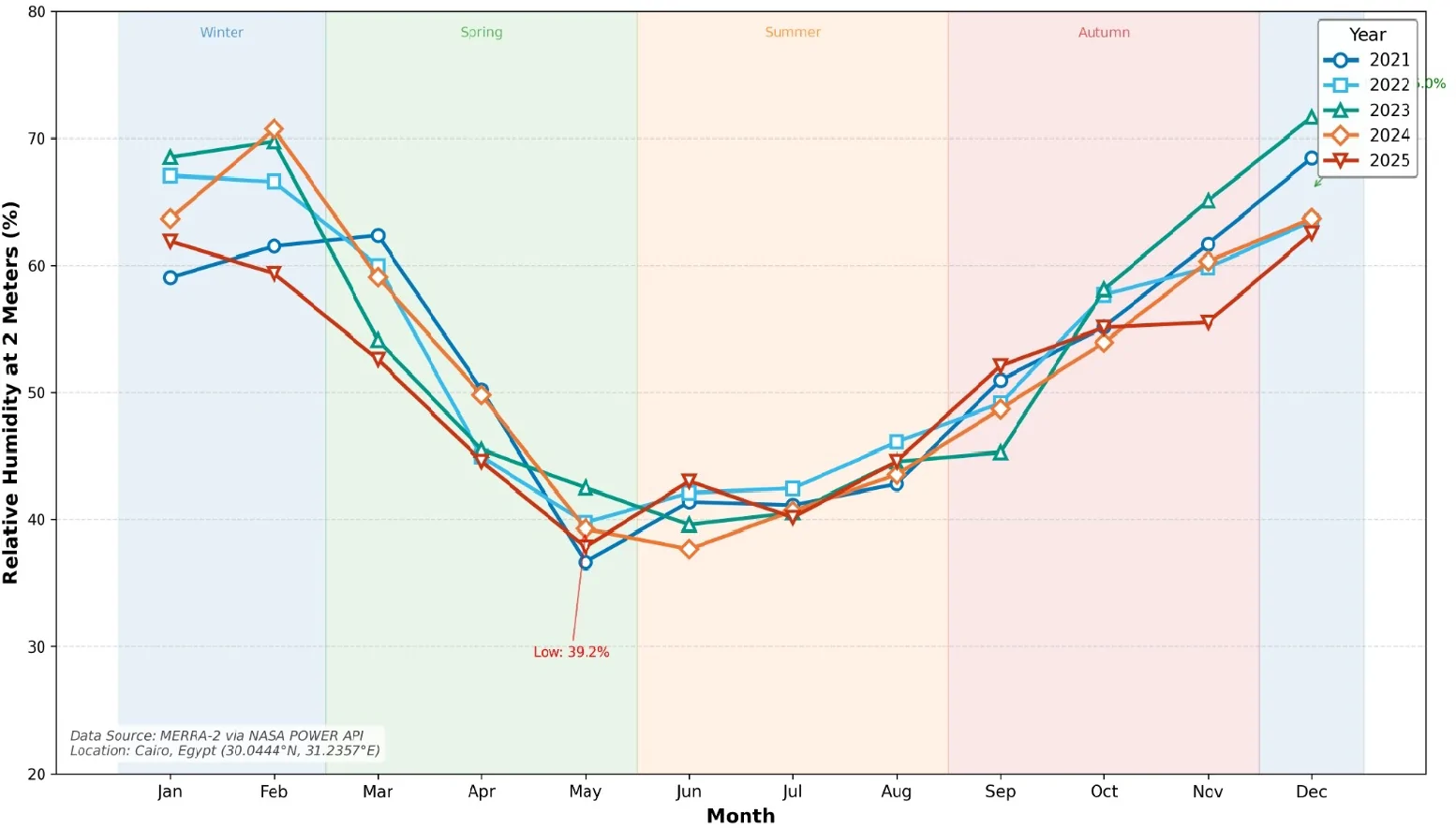
Monthly mean 2 m relative humidity time-series at Cairo (2021–2025) from MERRA-2 data. Recurring annual cycles and short-term fluctuations are evident; horizontal lines indicate annual mean values. The inter-annual stability of the seasonal minimum supports the adopted worst-case design condition.

The seasonal relative-humidity profile illustrated in Fig. 1 exhibits pronounced annual variability, with elevated moisture levels during the winter months (December–February) followed by a progressive decline from late spring through summer, reaching the seasonal minimum during May–June. Figure 2 presents the daily RH time series for the 2021–2025 period, confirming strong inter-annual consistency of the seasonal minimum within the 37–43% range. Accordingly, the selected design condition of RH = 38.19% constitutes a conservative worst-case operating scenario, expected to prevail only during the most thermally and hygrometrically demanding 2–3 months of the annual operating cycle.

#### Extended climatic record and inter-annual sensitivity

To address the statistical robustness of the adopted worst-case design condition, the MERRA-2 record for Cairo was extended beyond the initial 2021–2022 window to the full 2021–2025 period (Figs. 1 and 2). Across this five-year record, the minimum monthly mean relative humidity consistently occurs in May–June and remains confined to a narrow 37–43% band, with a five-year mean seasonal minimum of approximately 39.4% and a standard deviation below 2% points. The design value of RH = 38.19% therefore lies close to the lower bound of the observed inter-annual distribution and constitutes a conservative rather than an anomalous selection.

A sensitivity analysis was performed across the full observed range to quantify the propagation of inter-annual humidity variability into the sizing outcome. Because the required sponge area scales inversely with the concentration driving force ΔC (Eqs. 7–11), an increase in design relative humidity from 37% to 43% enlarges ΔC by approximately 18%, correspondingly reducing the required total exposed sponge area from about 156 cm² to about 132 cm², that is, from roughly + 5% to roughly − 11% relative to the 149 cm² baseline. The four-module geometry adopted herein (6.21 × 6.21 × 1.5 cm) therefore remains valid across the entire observed inter-annual envelope, with the baseline design retaining a positive area margin at the driest recorded condition. This bounded sensitivity confirms that the principal conclusions of the study are insensitive to the length of the reanalysis record employed.

#### Particulate loading and desert air quality considerations

The mass-transfer model formulated in Sect. 3.3 assumes moisture uptake from clean, particulate-free air, consistent with the controlled laboratory conditions under which the underlying water-uptake correlation was calibrated. Desert and peri-desert environments, however, are characterised by elevated aerosol loading, with Cairo experiencing recurrent khamsin dust episodes during the March–May period that coincides with the worst-case humidity window identified above. Two distinct degradation mechanisms are anticipated. First, deposition of hydrophilic and mineral dust on the exposed sponge faces progressively occludes the vapour-accessible porosity, reducing the effective exposed area (A_total) and hence the achievable water-uptake rate for a fixed concentration driving force. Second, alkaline-earth-rich mineral particulates (predominantly calcite and dolomite in the Egyptian Western Desert aerosol) may react with the sulphuric acid electrolyte to form sparingly soluble sulphate precipitates, which can partially passivate the electrode–electrolyte interface and gradually consume free acid.

The present analysis does not attempt to quantify these effects, as no dust-exposure experiments were performed and no validated deposition-kinetics data exist for the specific glass-foam matrix employed. Rather than introducing an unsupported correction factor, the results reported herein are explicitly framed as clean-air upper-bound estimates, and the following practical mitigation hierarchy is proposed for field deployment: (i) a replaceable coarse pre-filter of G4 class or equivalent upstream of the module intake faces, which removes the coarse fraction responsible for the majority of surface obstruction at a negligible vapour-transport penalty; (ii) a downward-facing intake geometry that exploits gravitational settling to limit direct impaction on the sponge faces; and (iii) a scheduled inspection and surface-cleaning interval synchronised with the 16.4 h electrolyte renewal cycle established in Sect. 3.3.3, such that particulate management imposes no additional maintenance. Experimental quantification of the dust-loading penalty under controlled aerosol concentrations is identified as a priority objective for the pilot-scale campaign outlined in Sect. 11.

### System architectures

**Fig. 3:**
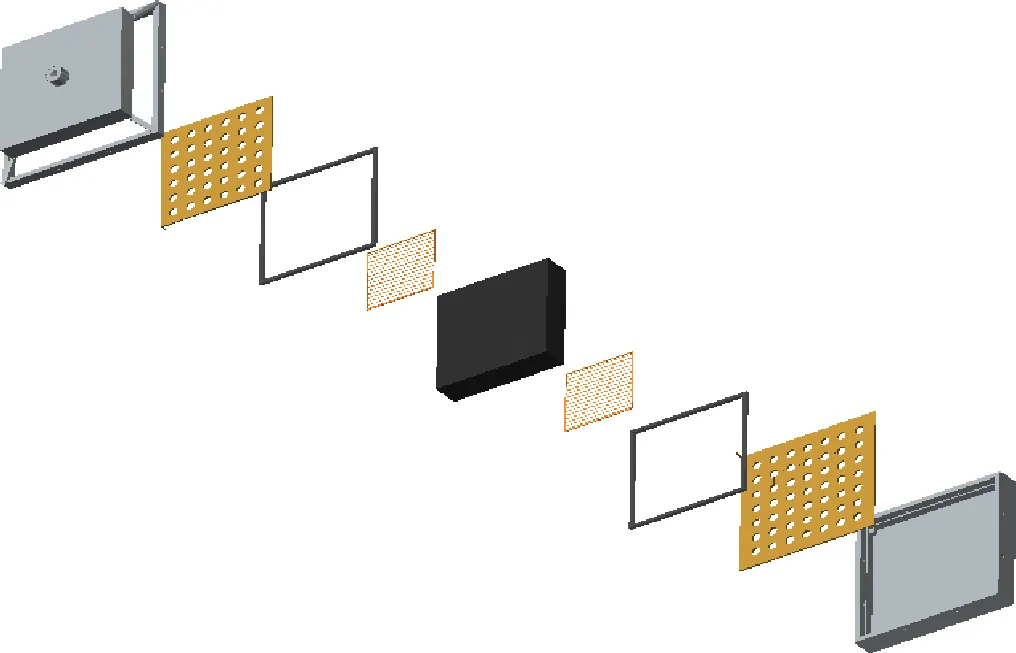
Exploded-view CAD assembly of the DAE module prototype. Components (left to right): end plate, anode current collector (perforated brass), air gasket, SS-904 L mesh electrode, glass-foam sponge matrix, SS-904 L mesh electrode, air gasket, cathode current collector, end plate. The bilaterally symmetric design ensures uniform moisture ingress from both exposed faces.

**Fig. 4:**
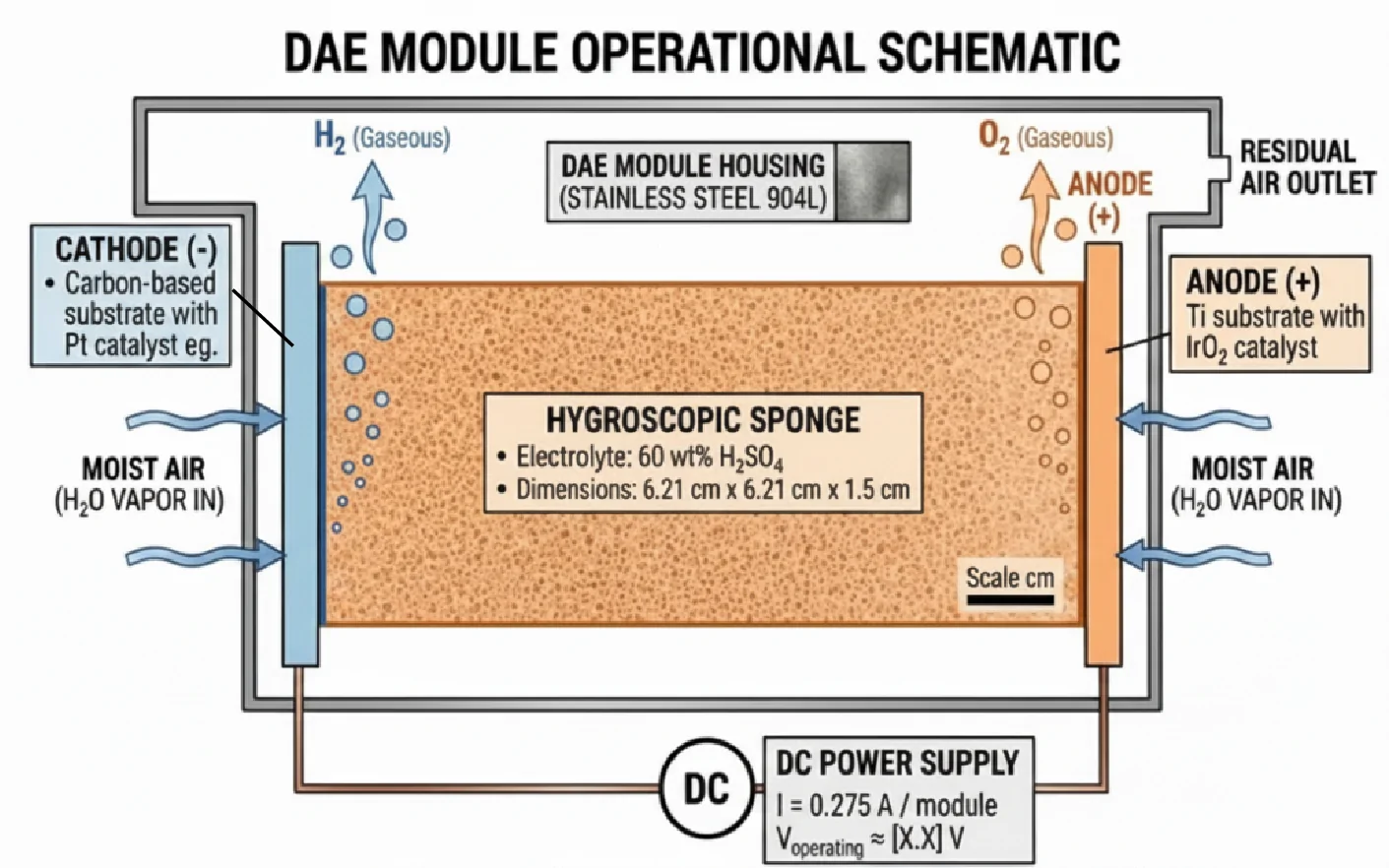
Cross-sectional schematic of a single DAE module showing the hygroscopic sponge saturated with 60 wt% H₂SO₄, bilateral moist-air intake, and embedded SS-904 L mesh electrodes. Four identical modules (6.21 × 6.21 × 1.5 cm) are connected in parallel to produce 0.5 L·h⁻¹ of H₂.

Two atmospheric-moisture-based hydrogen-production architectures are examined through a rigorous comparative investigation. The Direct Air Electrolyzer (DAE) module (Figs. 3 and 4) comprises an acid-resistant porous glass-foam matrix saturated with 60 wt% H₂SO₄, sandwiched between two Grade 904 L stainless-steel mesh electrodes. The exposed sponge surfaces absorb atmospheric moisture through hygroscopic uptake. Upon equilibration of the electrolyte with the prevailing ambient relative humidity, electrochemical water splitting proceeds at the embedded electrodes, yielding hydrogen at the cathode and oxygen at the anode. Four identical modules, each with external dimensions of 6.21 × 6.21 × 1.5 cm, are interconnected in a monopolar parallel configuration to achieve the target hydrogen-production rate of 0.5 L·h⁻¹.

**Fig. 5:**
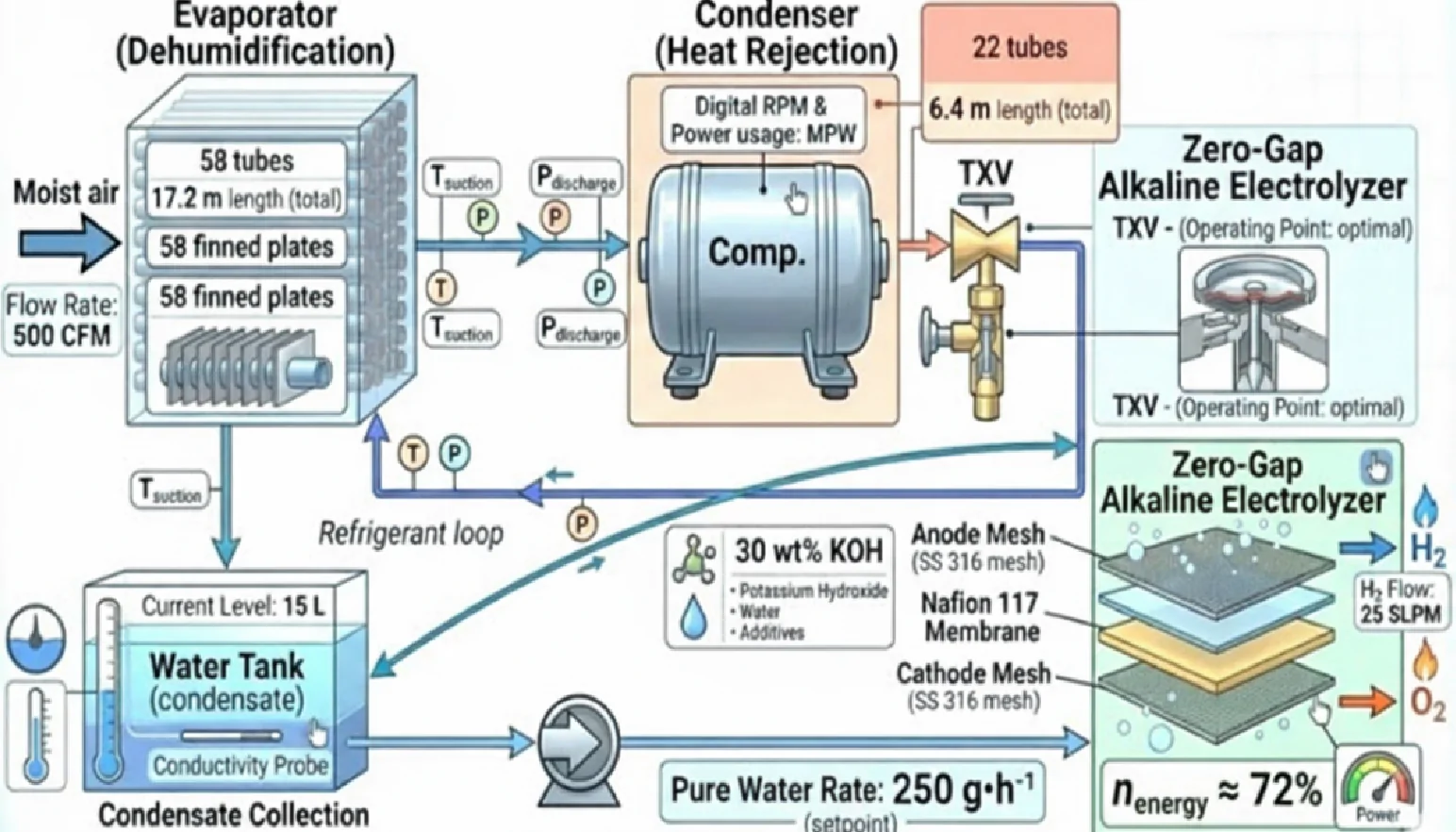
Schematic of the vapour-compression refrigeration cycle in the DH-AE system. Moist air enters the evaporator where it is cooled below the dew point; condensate is collected and fed to the alkaline electrolyzer. Dry exhaust air from the evaporator outlet is recirculated to the condenser inlet, improving cycle COP by approximately 12%.

In the hybrid DH-AE architecture (Fig. 5), ambient moist air is first cooled below its dew-point temperature by a compact vapour-compression refrigeration unit. The resulting condensate is collected in an intermediate storage reservoir and subsequently supplied to a zero-gap alkaline electrolyzer equipped with a Nafion 117 membrane and polished Grade 316 stainless-steel mesh electrodes immersed in a 30 wt% KOH electrolyte solution. The hot, dry exhaust air leaving the evaporator is recirculated to the condenser inlet. This heat-recovery configuration increases the condenser inlet air temperature by approximately 10 °C, resulting in an estimated 12% improvement in refrigeration-cycle coefficient of performance (COP) relative to the non-recuperative configuration.

These concerns are mitigated in the present design through acid-resistant Grade 904 L stainless steel, PTFE gaskets, and a soda–lime glass housing, and are bounded by an over-temperature interlock (Sect. 5.5) that keeps the electrolyte within the SS-904 L iso-corrosion envelope. However, long-term sealing integrity and corrosion durability remain open engineering challenges, and less-corrosive hygroscopic electrolytes (e.g., potassium acetate, lithium chloride, and deep eutectic solvents) are identified as priority alternatives in Sect. 11.

## Design Methodology

### Water-electrolysis thermodynamics

The overall liquid-phase water-electrolysis reaction is given by:

1 $$\:\begin{array}{cccc}&\:{\mathrm{H}}_{2}\mathrm{O}\left(\mathrm{l}\right)\to\:{\mathrm{H}}_{2}\left(\mathrm{g}\right)+\frac{1}{2}{\mathrm{O}}_{2}\left(\mathrm{g}\right)&\:&\end{array}$$

Under standard-state conditions (T° = 298 K and P° = 1 bar), the corresponding thermodynamic properties are ΔH° = +285.84 kJ·mol⁻¹, ΔS° = +163.15 J·mol⁻¹·K⁻¹, and ΔG° = +237.22 kJ·mol⁻¹^9^. The reversible and thermoneutral cell voltages are therefore expressed as:

2 $$\:\begin{array}{cccc}&\:{V}_{rev}=\frac{{\Delta\:}{G}^{\circ\:}}{zF}=1.229\:\mathrm{V}&\:&\:\end{array}$$

3 $$\:\begin{array}{cccc}&\:{V}_{tn}=\frac{{\Delta\:}{H}^{\circ\:}}{zF}=1.481\:\mathrm{V}&\:&\:\end{array}$$

where * z=2* is the number of electrons transferred per water molecule and $\:F=\mathrm{96,485}\:\mathrm{C}{\hspace{0.17em}}\mathrm{m}\mathrm{o}{\mathrm{l}}^{-1}$is Faraday’s constant. The actual operating cell voltage includes reversible, ohmic, and activation overpotential contributions:

4 $$\:\begin{array}{cccc}&\:{V}_{cell}={V}_{rev}+{V}_{ohmic}+{V}_{act}&\:&\end{array}$$

Concentration overpotentials were considered negligible given the relatively low operating current density employed in this study.

### Climate-based design-point humidity

The design-point relative humidity was determined using the NASA POWER MERRA-2 reanalysis dataset for Cairo, Egypt (30.06°N, 31.22°E) at a reference height of 2 m^32^. The minimum monthly mean relative humidity occurred in May of both years, recording values of 36.69% in 2021 and 39.69% in 2022. Accordingly, the design relative humidity was defined as the arithmetic mean of these two minima:

5 $$\:\begin{array}{cccc}&\:R{H}_{design}=\frac{36.69+39.69}{2}=38.19\mathrm{\%}&\:&\:\end{array}$$

This worst-case design condition is anticipated to prevail only during the most environmentally demanding 2–3 months of a typical operating year (Figs. 1 and 2). Based on the H₂SO₄–H₂O vapour-pressure isotherm at 25 °C^13^, a relative humidity of 38.19% corresponds to an equilibrium sulfuric acid concentration of $\:{X}_{eq}^{*}=49.305\:\mathrm{w}\mathrm{t}\mathrm{\%}$, as determined by linear interpolation of tabulated equilibrium data. An initial electrolyte concentration of 60 wt% H₂SO₄ was selected to sustain a sufficiently strong hygroscopic moisture-absorption driving force throughout the entire operating range.

Because the design condition is derived from a two-year MERRA-2 record (2021–2022), a sensitivity assessment was performed across the inter-annual minimum-humidity range of 37–43% observed over the extended 2021–2025 series (Fig. 2). Within this band, the adopted value of RH = 38.19% lies near the lower (most demanding) bound; the corresponding equilibrium concentration and required sponge area vary by less than approximately ± 8% across the range, confirming that the sizing outcome is robust to the limited observation window. Extension to a 5–10 year reanalysis record is on the other hand recommended to further improve the statistical robustness of the worst-case design definition, and is noted among the study limitations (Sect. 9.3).

**Fig. 6:**
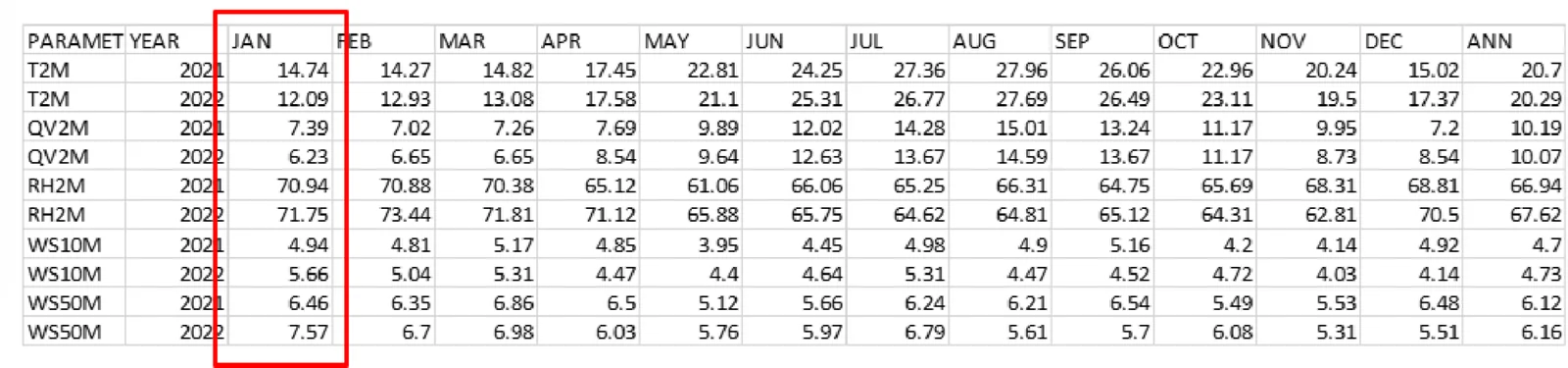
Climatic conditions and design parameters for El Dabaa, Egypt, based on NASA POWER data (2022), highlighting the variation in relative humidity and its role in defining the system worst-case operating condition.

To ensure realistic system design and performance evaluation, site-specific climatic data were incorporated into the analysis. The selected location is El Dabaa, Matrouh, Egypt, which represents a typical arid coastal environment within the MENA region. Meteorological data for the year 2022 were obtained from the NASA Prediction of Worldwide Energy Resources (POWER) database as shown in Fig. 6, including temperature, relative humidity, and wind speed parameters.

Based on this worst-case scenario, the required condensate production rate and system operating parameters were defined to maintain continuous hydrogen generation. The system is designed to produce 120 kg of hydrogen per day, corresponding to a water demand of approximately 1 m³ per day under continuous 24-hour operation.

This climate-driven design approach enables a realistic assessment of system feasibility and distinguishes the present work from conventional studies that assume ideal or constant environmental conditions.

### DAE Module Sizing

#### Mass and Molar Flow Rates

The DAE stack was dimensioned to achieve a target hydrogen volumetric flow rate of $\:{\dot{V}}_{{H}_{2}}=0.5\:\mathrm{L}{\hspace{0.17em}}{\mathrm{h}}^{-1}$under standard conditions (1 atm and 25 °C). Using the ideal gas relation:

6 $$\:\begin{array}{cccc}&\:{\dot{n}}_{{H}_{2}}=\frac{P{\dot{V}}_{{H}_{2}}}{RT}&\:&\:\end{array}$$

where $\:R=0.082057\:\mathrm{L}{\hspace{0.17em}}\mathrm{a}\mathrm{t}\mathrm{m}{\hspace{0.17em}}\mathrm{m}\mathrm{o}{\mathrm{l}}^{-1}{\hspace{0.17em}}{\mathrm{K}}^{-1}$, the hydrogen molar production rate is calculated as:

$$\:{\dot{n}}_{{H}_{2}}=\frac{\left(1\left)\right(0.5\right)}{\left(0.082057\left)\right(298\right)}=0.02046\:\mathrm{m}\mathrm{o}\mathrm{l}{\hspace{0.17em}}{\mathrm{h}}^{-1}$$

Based on the stoichiometry of Eq. (1), the corresponding mass flow rates are:

$$\:{\dot{m}}_{{H}_{2}O}=0.3686\:\mathrm{g}{\hspace{0.17em}}{\mathrm{h}}^{-1}$$ $$\:{\dot{m}}_{{H}_{2}}=0.04125\:\mathrm{g}{\hspace{0.17em}}{\mathrm{h}}^{-1}$$ $$\:{\dot{m}}_{{O}_{2}}=0.3274\:\mathrm{g}{\hspace{0.17em}}{\mathrm{h}}^{-1}$$

corresponding to the water-consumption rate, hydrogen-production rate, and oxygen co-production rate, respectively.

#### Concentration Driving Force and Required Sponge Area

The concentration driving force governing hygroscopic moisture uptake is determined by converting weight-fraction compositions into molar concentrations according to:

$$\:C=\frac{{X}_{wt}{\hspace{0.17em}}{\rho\:}_{soln}}{{M}_{{H}_{2}O}}$$

where $\:{X}_{wt}$is the water mass fraction, $\:{\rho\:}_{soln}$is the solution density (kg·m⁻³ or g·L⁻¹, consistently applied), and $\:{M}_{{H}_{2}O}$is the molar mass of water.

Substituting the corresponding values:

7 $$\:{C}_{0}=\frac{0.40\times\:1494\times\:{10}^{3}}{18.015}=\mathrm{33,172}\:{\mathrm{mol.m}}^{-3}\:$$

(initial condition: 60 wt% H₂SO₄).

8 $$\:{C}^{*}=\frac{0.5070\times\:1384\times\:{10}^{3}}{18.015}=\mathrm{38,957}\:{\mathrm{mol.m}}^{-3}$$

(equilibrium at RH = 38.19%).

9 $$\:{\Delta\:}C={C}^{*}-{C}_{0}=\mathrm{38,957}-\mathrm{33,172}=\mathrm{5,785}\:{\mathrm{mol.m}}^{-3}$$

The minimum total exposed sponge area required to sustain the target water-uptake rate via molecular diffusion into the hygroscopic electrolyte is obtained using Fick’s first law^15^:

10 $$\:{\dot{m}}_{{H}_{2}O}={M}_{{H}_{2}O}{\hspace{0.17em}}\widehat{k}{\hspace{0.17em}}{A}_{total}{\hspace{0.17em}}{\Delta\:}C\:\:$$

where $\:\widehat{k}=6.6*{10}^{-8}{\hspace{0.17em}}\mathrm{m/s}$ the conservative (worst-case) mass-transfer coefficient for water vapour diffusion into 60 wt% H₂SO₄ at 25 °C, calibrated using experimental water-uptake data reported by Guo et al.^15^.

A conservative reduction in effective absorption area on the order of 10–25% may be expected under sustained desert dust loading conditions, depending on particle size distribution and exposure duration. This estimate is derived from preliminary fouling factors observed in similar air-exposed sorbent systems operating in arid climates (e.g., El-Dabaa, Egypt; see Sect. 9 Real Case Study). Periodic surface cleaning and hydrophobic pre-filter meshes are recommended as practical mitigation measures, while quantitative characterization of dust-induced mass-transfer degradation is identified as a priority for future experimental work.

Rearranging Eq. (10) yields:

$$\:{A}_{total}=\frac{{\dot{m}}_{{H}_{2}O}}{{M}_{{H}_{2}O}{\hspace{0.17em}}\widehat{k}{\hspace{0.17em}}{\Delta\:}C}$$

Substituting numerical values gives:

11 $$\:{A}_{total}=\frac{1.024\times\:{10}^{-4}}{18.015\times\:6.6\times\:{10}^{-8}\times\:\mathrm{5,785}}=149\:{\mathrm{cm}}^{2}\:$$

The total active area is distributed across $\:n=4\:$identical monopolar modules. Each sponge is assumed to be a cuboid of square cross-section $\:X\times\:X$cm and thickness 1.5 cm. Each module exposes four lateral faces, yielding an effective area:

$$\:{A}_{module}=4\times\:\left(1.5X\right)\times\:2=12X$$

Thus, enforcing $\:n{A}_{module}={A}_{total}$gives:

$$\:4\times\:12X=149$$

which yields:

$$\:X=6.21\mathrm{\:cm}$$

Therefore, each sponge has dimensions $\:6.21\times\:6.21\times\:1.5{\hspace{0.17em}cm}$.

The selected sponge thickness of 1.5 cm represents an experimentally validated optimum: increasing the thickness leads to a significant rise in ohmic resistance (Sect. 6.2), whereas reducing it diminishes the electrolyte volume and shortens the replenishment interval, thereby compromising both system stability and operational autonomy.

#### Absorption Time to Equilibrium

The volume of 60 wt% H₂SO₄ electrolyte retained within a single sponge is assumed equal to the sponge geometric volume:

$$\:{V}_{\mathrm{sponge}}=6.21\times\:6.21\times\:1.5=57.85\:{\mathrm{cm}}^{3}$$

The corresponding initial and equilibrium water masses within this volume are $\:{m}_{\mathrm{water},0}=34.57\mathrm{\:g}$and $\:{m}_{\mathrm{water,eq}}=40.60\mathrm{\:g}$, respectively. The maximum electrolyte replenishment interval under worst-case ambient humidity conditions is therefore estimated as:

12 $$\:\begin{array}{cccc}&\:{t}_{\mathrm{absorption}}=\frac{{m}_{\mathrm{water,eq}}-{m}_{\mathrm{water},0}}{{\dot{m}}_{{\mathrm{H}}_{2}\mathrm{O}}}=\frac{40.60-34.57}{0.369}=16.4\mathrm{\:h}&\:&\:\end{array}$$

Under more favourable humidity conditions (RH > 50%), the water uptake rate increases, leading to a proportional extension of the service interval.

### DAE Voltage and Efficiency

#### Required Current and Electrode Sizing

The total electrical current required to sustain the target hydrogen production rate is derived from Faraday’s law of electrolysis:

13 $$\:\begin{array}{cccc}&\:{I}_{\mathrm{total}}=\frac{{\dot{m}}_{{\mathrm{H}}_{2}}{\hspace{0.17em}}F}{Z}=\frac{0.04125\times\:\mathrm{96,485}}{1.008\times\:3600}=1.10\mathrm{\:A}&\:&\end{array}$$

where * F*is the Faraday constant (96,485 C·mol⁻¹) and $\:Z\:$represents the electrochemical equivalent of hydrogen.

$$\:Z=\frac{{M}_{{\mathrm{H}}_{2}}}{{n}_{e}}=\frac{2.016}{2}=1.008\:{{g\cdot\:mol}}^{-1}\cdot{\mathrm{electron}}^{-1}$$

For a four-module stack operating in parallel, the per-module current is:

14 $$\:\begin{array}{cccc}&\:{I}_{\mathrm{module}}=\frac{{I}_{\mathrm{total}}}{4}=\frac{1.10}{4}=0.275\mathrm{\:A}&\:&\:\end{array}$$

Assuming a steady-state current density of $\:J=15\:{{mA\cdot\:cm}}^{-2}$, determined experimentally from polarization measurements of SS-904 L mesh electrodes in 60 wt% H₂SO₄^15^, the required electrochemically active electrode area per module is calculated as:

15 $$\:\begin{array}{cccc}&\:{A}_{\mathrm{active}}=\frac{{I}_{\mathrm{module}}}{J}=\frac{0.275}{0.015}=18.33\:{\mathrm{cm}}^{2}&\:&\:\end{array}$$

#### Voltage Decomposition

The ohmic overpotential is evaluated from the combined electrical resistances of the electrolyte and electrode components. For a 60 wt% H₂SO₄ electrolyte with an electrical resistivity of $\:{\rho\:}_{e}=2.68\:{\Omega\:}\cdot\:\mathrm{cm}$, a conduction path length equal to the sponge thickness ($\:L=1.5\mathrm{\:cm}$), and a cross-sectional area $\:A={6.21}^{2}=38.6\:{\mathrm{cm}}^{2}$, the electrolyte resistance is given by:

16 $$\:\begin{array}{cccc}&\:{R}_{\mathrm{electrolyte}}={\rho\:}_{e}\frac{L}{A}=2.68\times\:\frac{1.5}{38.6}=0.104\:{\Omega\:}&\:&\:\end{array}$$

For the SS-904 L mesh electrode (resistivity $\:\rho\:=95.2\:\mu\:{\Omega\:}\cdot\:\mathrm{cm}$, thickness $\:1\mathrm{\:mm}$, and active area $\:{A}_{\mathrm{active}}=18.33\:{\mathrm{cm}}^{2}$), the electrode resistance is on the order of $\:\sim\:{10}^{-7}\:{\Omega\:}$, and is therefore negligible compared with the electrolyte contribution. Consequently, the ohmic overpotential is approximated as:

17 $$\:\begin{array}{cccc}&\:{V}_{\mathrm{ohmic}}={I}_{\mathrm{module}}\left({R}_{\mathrm{electrolyte}}+{R}_{\mathrm{electrode}}\right)\approx\:0.275\times\:0.104=0.029\mathrm{\:V}&\:&\:\end{array}$$

The activation overpotential is determined using the empirical Tafel-type correlation reported by Guo et al.^13^ for the H₂SO₄-based DAE system:

18 $$\:{V}_{\mathrm{act}}=s\cdot\:\mathrm{l}\mathrm{o}\mathrm{g}\left[\left(\frac{t}{{A}_{\mathrm{active}}}\right){I}_{\mathrm{module}}+1\right]\:\begin{array}{cccc}&\:=0.185\cdot\:\mathrm{l}\mathrm{o}\mathrm{g}\left[\left(\frac{1.002}{18.33\times\:{10}^{-4}}\right)\times\:0.275+1\right]=0.403\mathrm{\:V}&\:&\:\end{array}$$

The Tafel parameters adopted in the present study were derived from the platinum-mesh DAE experiments reported by Guo et al.^15^. Since the fabricated prototype employs SS-904 L stainless-steel electrodes, which exhibit lower catalytic activity for hydrogen evolution than platinum, the calculated activation overpotential should be interpreted as a lower-bound estimate.

Based on reported hydrogen-evolution overpotential differences between platinum and austenitic stainless steels in acidic electrolytes, the actual operating voltage of the SS-904 L configuration is anticipated to exceed the model prediction by approximately 80–140 mV under comparable current densities. This correction remains consistent with the experimentally measured operating voltage range reported in Sect. 8.

Accordingly, the present analysis is best interpreted as a bounded design estimate intended to characterize comparative system behaviour rather than to provide a precise electrochemical prediction.

##### Electrode-Material Correction and Reconciliation with Measured Voltage

Because the discrepancy between the platinum-calibrated model and the stainless-steel prototype is central to the interpretation of all efficiency metrics reported in this work, the correction is formalised here rather than treated qualitatively. The activation overpotential of Eq. (18) is decomposed into a platinum-referenced term and an additive material-transfer penalty:

$$\:{\mathbf{V}}_{\mathbf{a}\mathbf{c}\mathbf{t},\mathbf{S}\mathbf{S}-904\mathbf{L}}=\:{\mathbf{V}}_{\mathbf{a}\mathbf{c}\mathbf{t},\mathbf{P}\mathbf{t}}+\:{\varDelta\:\mathbf{V}}_{\mathbf{H}\mathbf{E}\mathbf{R}},\:\:\:\:\:\:\mathbf{w}\mathbf{i}\mathbf{t}\mathbf{h}\:{\varDelta\:\mathbf{V}}_{\mathbf{H}\mathbf{E}\mathbf{R}}\:=\:0.080-0.140\:\mathbf{V}$$

where ΔV_HER denotes the additional hydrogen-evolution overpotential of austenitic stainless steel relative to platinum mesh in concentrated acidic electrolyte at comparable current density. Applying this bound to the platinum-referenced value of 0.403 V yields a corrected activation overpotential of 0.483–0.543 V and a corrected module operating voltage of:

$$\:{\mathbf{V}}_{\mathbf{D}\mathbf{A}\mathbf{E},\mathbf{S}\mathbf{S}-904\mathbf{L}\:}=\:1.229\:+\:0.053\:+\:\left(0.483\:\mathbf{t}\mathbf{o}\:0.543\right)\:=\:1.766\:\mathbf{t}\mathbf{o}\:1.826\:\mathbf{V}$$

The measured steady-state prototype voltage of 1.82–1.95 V reported in Sect. 8.3 brackets the upper limit of this corrected prediction. The residual difference of approximately 0–125 mV between the corrected model and the measured range is attributable to contact resistance at the current-collector–mesh interface, incomplete and spatially non-uniform electrolyte saturation of the glass-foam matrix, and the fact that laboratory measurements were conducted at RH = 45–55% rather than at the equilibrium saturation state assumed by the model. The corrected model therefore reproduces the experimental behaviour within the expected uncertainty of a first-principles design calculation, whereas the uncorrected platinum-referenced model does not.

Two distinct sets of performance figures are consequently reported throughout this manuscript and must not be conflated. The platinum-benchmark projection (V = 1.686 V, $\:{\eta\:}_{\mathrm{energy}}=\:87.8\mathrm{\%}$) represents the performance attainable with a state-of-the-art noble-metal catalyst and defines the upper performance envelope of the DAE architecture. The SS-904 L realisable estimate (V = 1.766–1.826 V, $\:{\eta\:}_{\mathrm{energy}}=\:81.1-83.9\mathrm{\%}$) represents the performance of the low-cost, acid-compatible electrode actually employed in the prototype. All efficiency values quoted in the abstract, Sect. 3.4.3, and Sect. 6.1 refer to the platinum-benchmark projection and are explicitly labelled as such; the corresponding SS-904 L values are tabulated in Table 2 for direct comparison. This dual-basis study preserves the original design results while ensuring that the reported efficiencies are not misinterpreted as measured stainless-steel performance.

where $\:s=0.185\mathrm{\:V\:}$is the Tafel slope coefficient and $\:t=1.002\:{\mathrm{A}}^{-1}\cdot\:{\mathrm{m}}^{2}\:$is an experimentally derived exchange-current parameter^13^.

The total operating voltage of the DAE module is therefore:

19 $$\:\begin{array}{cccc}&\:{V}_{\mathrm{DAE}}={V}_{\mathrm{rev}}+{V}_{\mathrm{ohmic}}+{V}_{\mathrm{act}}=1.229+0.053+0.403=1.686\mathrm{\:V}&\:&\:\end{array}$$

The activation overpotential (0.403 V), constituting approximately 24% of the total cell voltage, represents the dominant loss mechanism. This finding indicates that electrode kinetics constitute the primary performance limitation under the prevailing operating conditions, rather than ionic conduction through the electrolyte. Accordingly, further efficiency gains are expected to be more effectively realised through targeted electrode catalyst enhancement than through modifications to electrolyte geometry or cell architecture.

#### Stack Power and Efficiency

The per-module and total stack power consumptions are calculated as:

$$\:{P}_{\mathrm{module}}={I}_{\mathrm{module}}\times\:{V}_{\mathrm{DAE}}=0.275\times\:1.686=0.464\mathrm{\:W}$$

$$\:{P}_{\mathrm{stack}}=4\times\:{P}_{\mathrm{module}}=4\times\:0.464=1.856\mathrm{\:W}$$

Two complementary performance metrics are employed to evaluate the electrochemical efficiency of the DAE system. The voltage-based energy efficiency is defined as:

20 $$\:\begin{array}{cccc}&\:{\eta\:}_{\mathrm{energy}}=\frac{{V}_{\mathrm{tn}}}{{V}_{\mathrm{DAE}}}=\frac{1.481}{1.686}=87.8\mathrm{\%}&\:&\:\end{array}$$

where $\:{V}_{\mathrm{tn}}$is the thermoneutral voltage of water electrolysis. In addition, the electrical utilization efficiency is expressed as:

21 $$\:\begin{array}{cccc}&\:{\eta\:}_{\mathrm{elec}}=\frac{{P}_{\mathrm{stack}}-{I}_{\mathrm{total}}{V}_{\mathrm{ohmic}}}{{P}_{\mathrm{stack}}}=\frac{1.856-(1.10\times\:0.053)}{1.856}=97.3\mathrm{\%}&\:&\:\end{array}$$

It is important to emphasise that the energy efficiency (87.8%) and electrical utilisation efficiency (97.3%) reported for the DAE configuration are cell-level metrics evaluated on a voltage basis and an ohmic-loss basis, respectively, and do not incorporate balance-of-plant auxiliary loads such as sensing, control, and safety actuation systems. When these auxiliary electrical loads are included, the aggregate DAE system efficiency is estimated at approximately 84–86%, compared with 68.5% for the DH-AE configuration. The voltage-based efficiency of 87.8% is therefore not directly comparable to higher-heating-value (HHV)-based efficiencies conventionally reported for alkaline electrolyzers without explicit normalisation to a common thermodynamic reference basis.

**Fig. 7:**
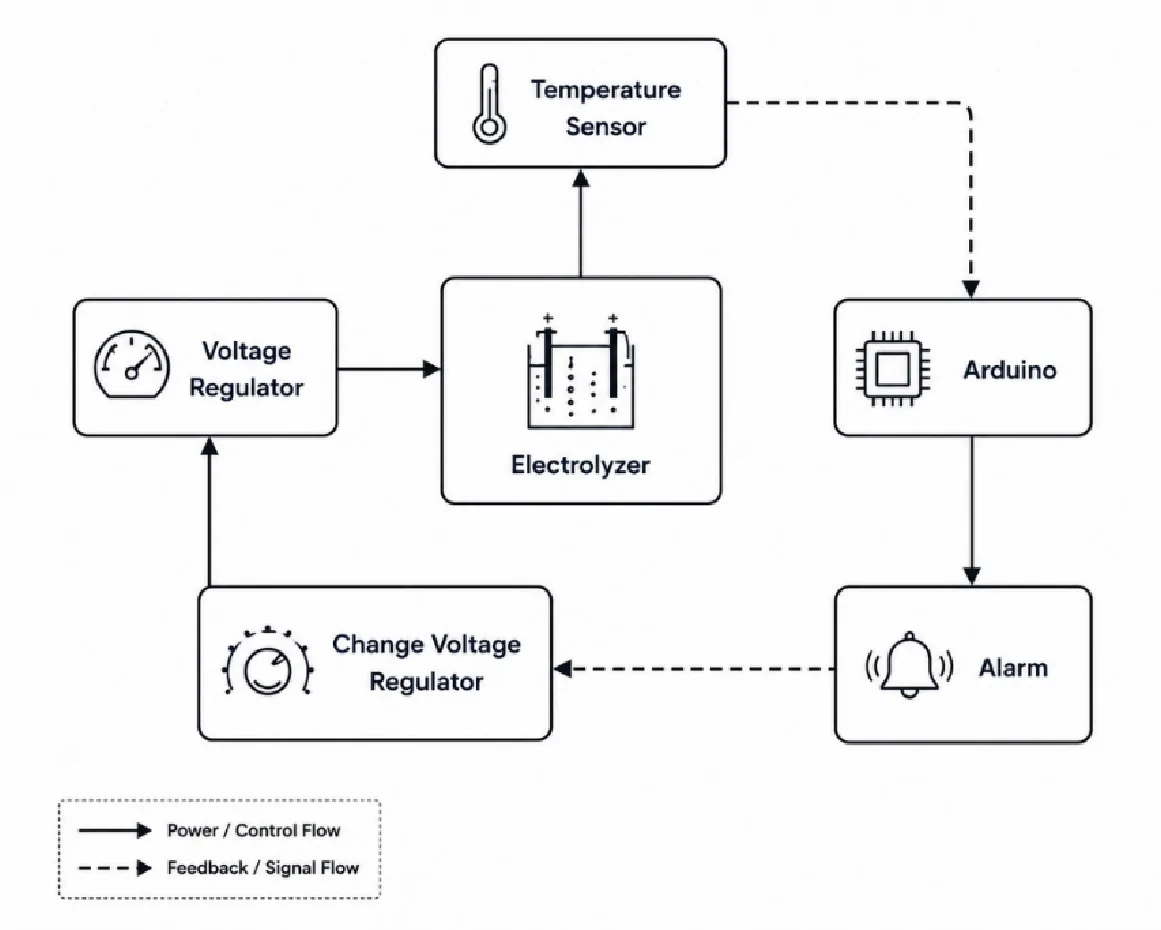
Temperature control and safety block diagram of the electrolyzer system with Arduino-based monitoring and control.

The overall temperature control and safety mechanism of the electrolyzer system is illustrated in Fig. 7, highlighting the integration of voltage regulation, real-time temperature monitoring, Arduino-based control, and an alarm feedback loop to ensure safe and efficient operation.

To maintain a clear distinction between the idealized electrochemical ceiling and the realistic performance of the fabricated device, Table 2 summarizes the key performance indicators on a dual basis: the platinum-benchmark projection (Sect. 3.4.2), the SS-904 L-corrected estimate (Sect. 3.4.3), and the experimentally measured prototype range (Sect. 8.3). The measured module voltage of 1.82–1.95 V falls slightly above the corrected band of 1.766–1.826 V; the residual difference of approximately 50–120 mV is attributable to contact resistance and non-uniform electrolyte distribution within the sponge matrix, rather than to a failure of the electrochemical model. Consequently, the SS-904 L-corrected energy efficiency of 81.1–83.9% represents the realistic design target for the present stainless-steel hardware, while the platinum benchmark of 87.8% defines the upper performance ceiling that could be approached through catalyst enhancement (e.g., platinum-group metal coatings or advanced HER catalysts). The electrical utilisation efficiency remains essentially unchanged across the three bases because the ohmic loss term is dominated by the electrolyte resistance, which is independent of electrode catalytic activity.

**Table 2:** Dual basis performance summary for the DAE module: platinum benchmark projection versus SS 904 L realisable estimate.

Parameter	Symbol	Pt Benchmark (Sect. 3.4.2)	SS-904 L Estimate (Sect. 3.4.3)	Measured (Prototype, Sect. 8.3)
Activation overpotential	$\:{V}_{\mathrm{act}}$	0.403 V	0.483–0.543 V	(measured, not separable)
Module operating voltage	$\:{V}_{\mathrm{DAE}}$	1.686 V	1.766–1.826 V	1.82–1.95 V
Voltage-basis energy efficiency	$\:{\eta\:}_{\mathrm{energy}}$	87.8%	81.1–83.9%	75.9–81.4%
Electrical utilisation efficiency	$\:{\eta\:}_{\mathrm{elec}}$	97.3%	97.1–97.2%	—
Stack power (4 modules)	Pt benchmark	1.856 W	1.943–2.009 W	—

The electrical utilisation efficiency $\:{\eta\:}_{\mathrm{elec}}$ is comparatively insensitive to electrode material because the ohmic loss term is dominated by ionic conduction through the acid-saturated sponge and is unaffected by catalyst identity; conversely, the voltage-basis efficiency $\:{\eta\:}_{\mathrm{energy}}$ shifts by approximately 4–7% points, which quantifies the value of catalytic enhancement identified in Sect. 6.1 as the highest-priority development pathway.

The parameter $\:{\eta\:}_{\mathrm{energy}}$quantifies the proximity of the operating cell voltage to the thermoneutral limit, whereas $\:{\eta\:}_{\mathrm{elec}}$represents the fraction of supplied electrical power effectively utilized for electrochemical conversion rather than dissipated through ohmic heating.

The high electrical utilisation efficiency is primarily attributable to the low ohmic resistance of the DAE configuration, which arises from the short ionic transport path within the acid-saturated sponge matrix and the elimination of auxiliary pumping requirements. Nevertheless, these efficiency values must be interpreted within the context of the present definitions and system boundary conditions. Direct comparison with the commonly reported 65–75% higher-heating-value (HHV)-based efficiencies of conventional alkaline electrolyzers requires careful normalization of the respective efficiency formulations and balance-of-system assumptions^33,34^.

**Fig. 8:**
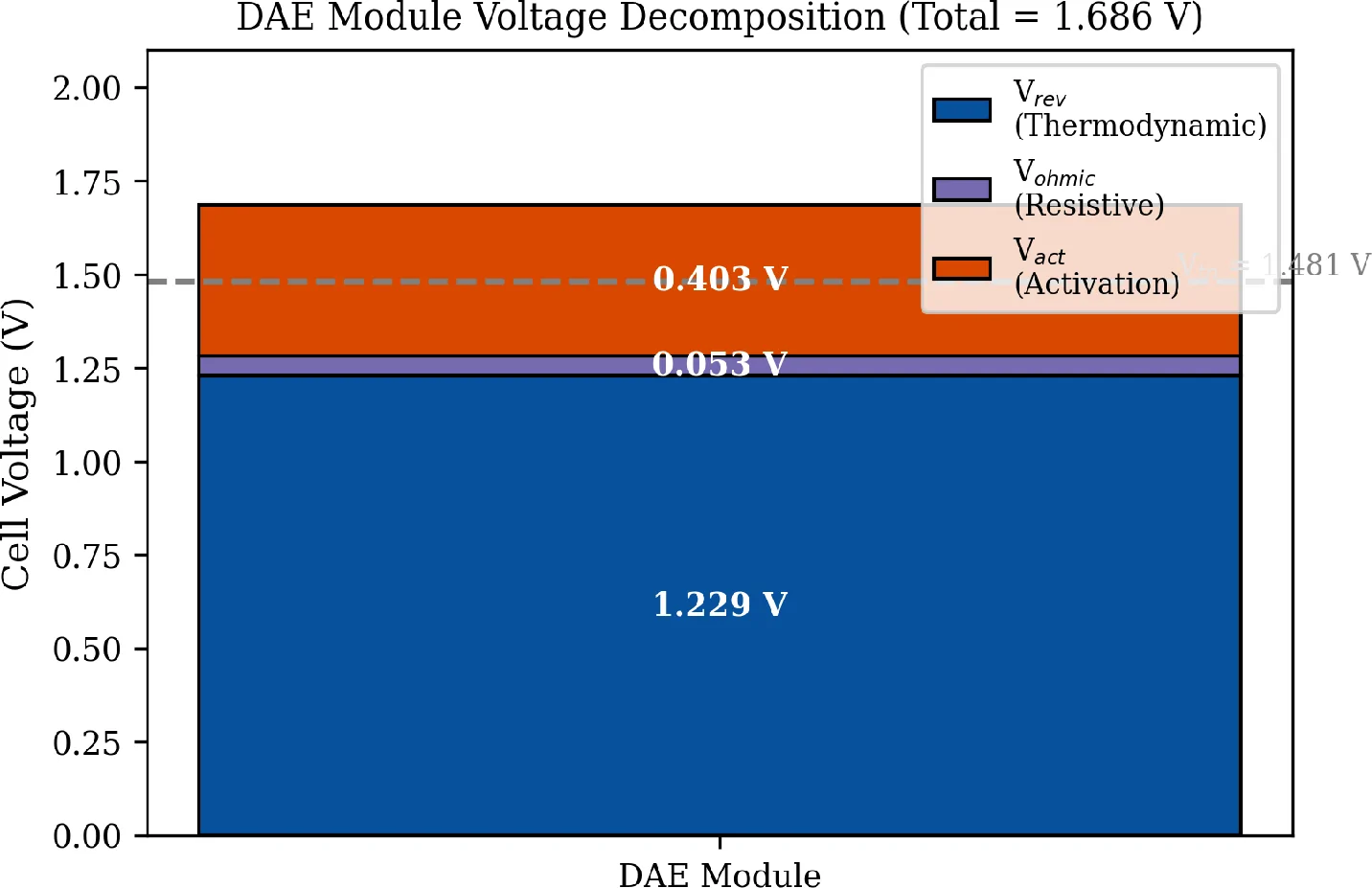
Voltage decomposition of the DAE module with thermoneutral voltage reference (V_tn_).

Figure 8 illustrates the voltage decomposition of the DAE module into reversible, ohmic, and activation components. It can be observed that the thermoneutral voltage (V_tn_ = 1.481 V) is exceeded primarily by the activation overpotential, indicating that the system operates close to the reversible limit.

### DH-AE system sizing

The DH-AE system is designed to produce a condensate flow rate of $\:250\:{\mathrm{g.h}}^{-1}$, corresponding to the water supply required to sustain a hydrogen production capacity equivalent to that of the DAE configuration. Under worst-case ambient conditions ($\:{T}_{\mathrm{db}}=25.13{\:}^{\circ\:}\mathrm{C}$, $\:{\omega\:}_{i}=5.13\:{\mathrm{g.kg}}^{-1}$dry air), the required dry-air mass flow rate is determined as:

22 $$\:\begin{array}{cccc}&\:{\dot{m}}_{\mathrm{da}}=\frac{{\dot{m}}_{w}}{{\omega\:}_{i}-{\omega\:}_{o}}=\frac{250}{5.13}=48.73\:{\mathrm{kg.\:h}}^{-1}&\:&\:\end{array}$$

where $\:{\omega\:}_{i}$and $\:{\omega\:}_{o}$denote the inlet and outlet humidity ratios, respectively.

The refrigeration subsystem is driven by a B38G hermetic compressor rated at 1/6 HP, delivering a cooling capacity of 297 W at a coefficient of performance (COP) of 2.87. The evaporator geometry was designed using the overall thermal-resistance approach, resulting in a configuration consisting of 58 tubes (outer diameter $\:9.525\mathrm{\:mm}$, inner diameter $\:8.125\mathrm{\:mm}$) with a total tube length of 17.2 m, in combination with 62 aluminium fins of dimensions $\:22\times\:4\times\:0.05\mathrm{\:cm}$.

**Fig. 9:**
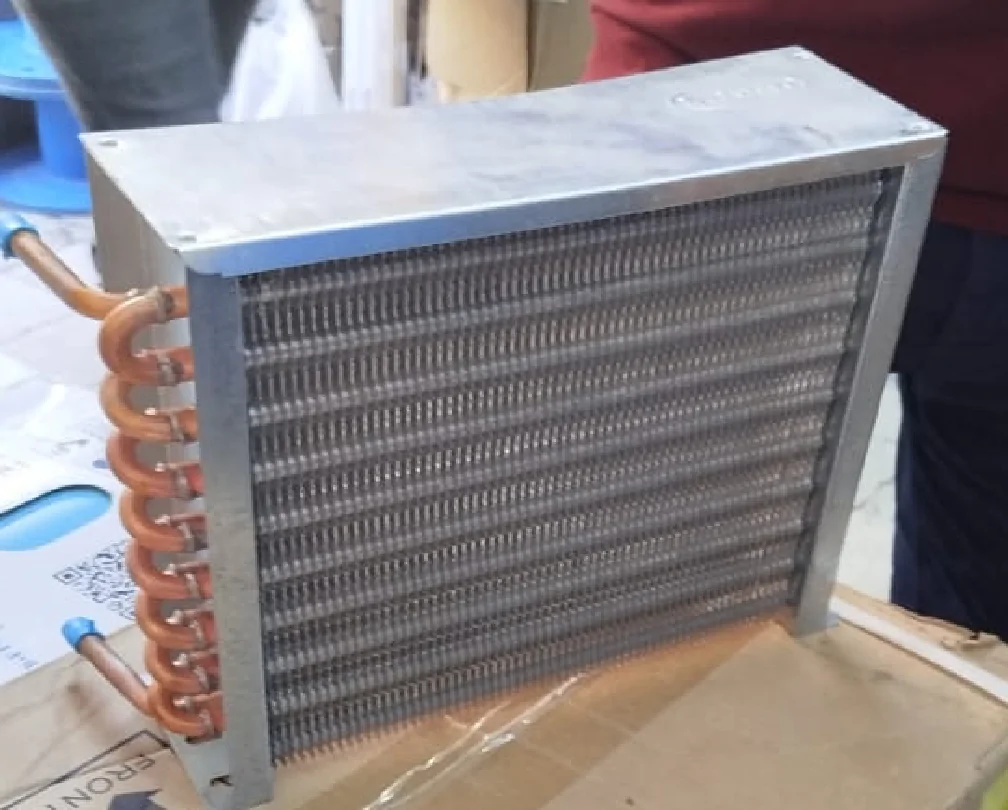
Finned-tube evaporator used for thermal regulation of the electrolyzer system.

To enhance thermal regulation of the electrolyzer system, a finned-tube evaporator is employed as illustrated in Fig. 9.

The condenser section comprises 22 tubes with a total tube length of 6.4 m. The performance of the alkaline electrolyzer subsystem was calibrated using the Aspen Plus model validated by Sánchez et al.^35^ and reported in the *International Journal of Hydrogen Energy* (IJHE).

Accounting for compressor power consumption, auxiliary fan demand, and pump loads, the integrated DH-AE system achieves an overall energy efficiency of 68.5%.

To provide a comprehensive comparison of the mass and energy performance between the two configurations, Table 3 summarizes the key design and operational parameters of the DAE and DH-AE architectures at their respective operating points.

**Table 3:** Comparative mass and energy balance for the DAE and DH-AE architectures at their respective design points.

Parameter	DAE	DH-AE
H₂ production rate	0.5 L·h⁻¹	~ 0.5 L·h⁻¹ (equivalent class)
Water source	Atmospheric moisture (direct)	Refrigeration condensate
Water consumption	0.369 g·h⁻¹ per module	250 g·h⁻¹ condensate
Electrolyte	60 wt% H₂SO₄	30 wt% KOH
Operating voltage	1.686 V per module	~ 1.9–2.1 V (AE stack)
Stack power	1.856 W	~ 3–5 W (AE) + compressor
η_energy_	87.8%	~ 72% (AE cell)
Aggregate system efficiency	97.3% (electrolysis)	68.5% (system incl. DH)
Temperature operating limit	38 °C (904 L isocorrosion)	Ambient (no acid constraint)

**Fig. 10:**
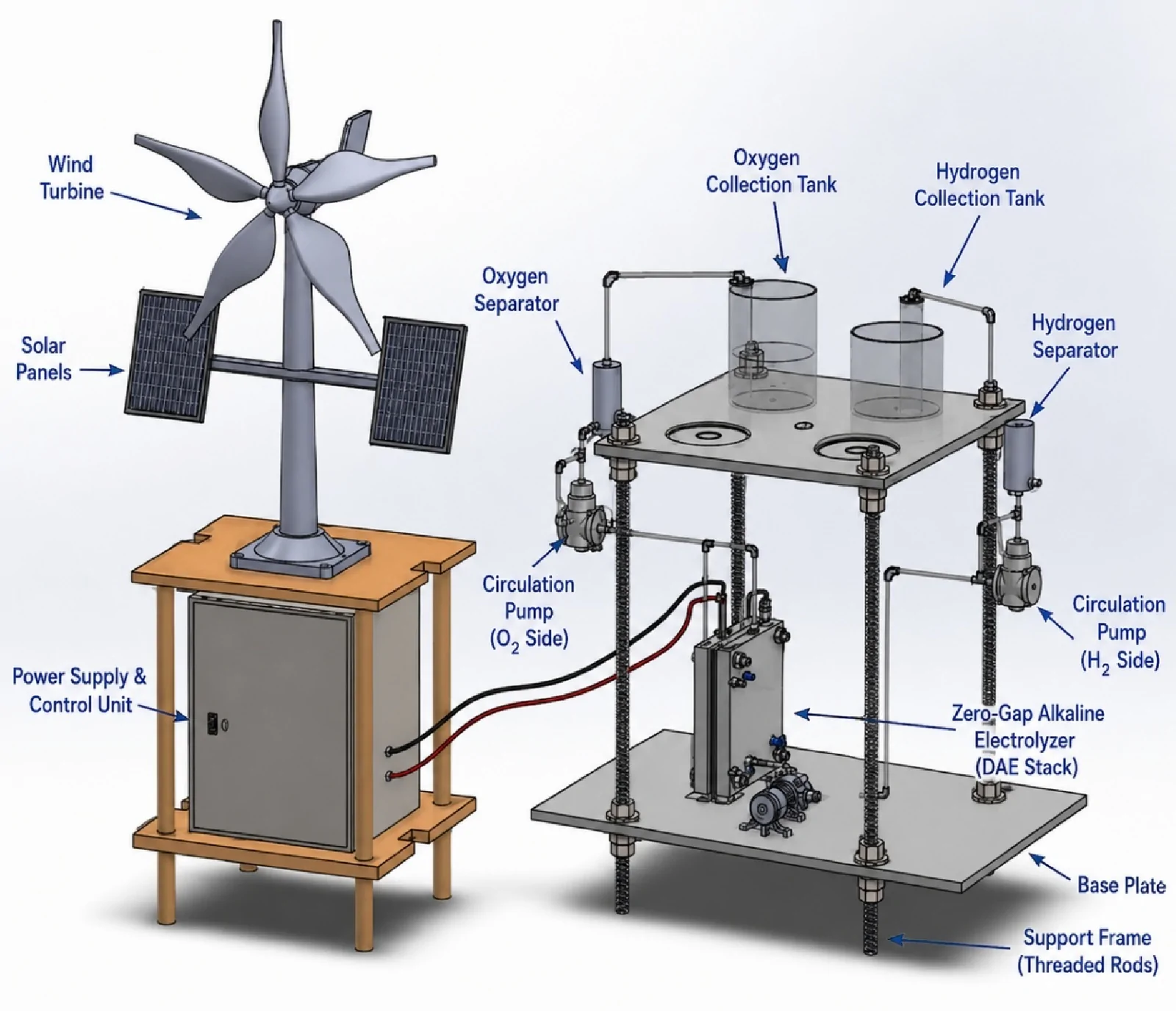
Experimental setup of the integrated renewable energy-driven zero-gap alkaline electrolyzer (DAE) system.

Figure 10 presents the experimental setup of the integrated renewable energy-driven zero-gap alkaline electrolyzer (DAE) system. The system is powered by a hybrid renewable energy source combining wind and solar energy, connected to a power supply and control unit. The electrolyzer stack is integrated with hydrogen and oxygen separation units, circulation pumps, and gas collection tanks. Additionally, a fluid handling structure is incorporated to ensure continuous electrolyte circulation and efficient gas production and separation.

## Numerical simulation and thermal validation

### Model description and boundary conditions

A three-dimensional steady-state thermal finite-element analysis (FEA) of the complete four-module DAE stack was conducted using ANSYS to: (i) verify that all components operate within acceptable thermal limits; (ii) assess the suitability of Grade 904 L stainless steel as the electrode material; and (iii) identify potential thermal hot spots within the stack assembly.

**Fig. 11:**
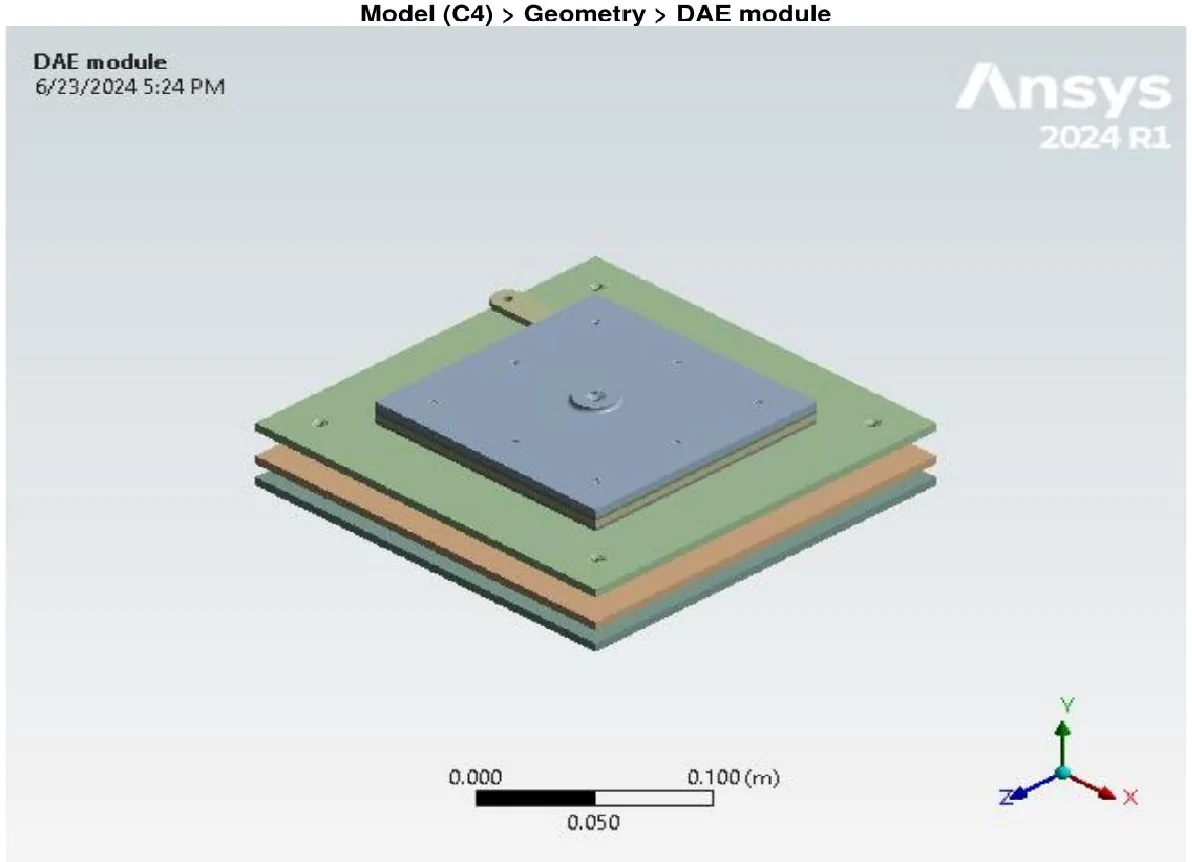
ANSYS 2024 R1 DAE module geometry imported from SolidWorks. The layered assembly shows (bottom to top): glass housing base (teal), acid-saturated sponge (brown), SS-904 L mesh electrode (grey), Teflon gasket (dark), upper SS-904 L electrode, and glass housing lid. Dimensions: 6.21 × 6.21 × 1.5 cm per module.

The numerical model geometry (Fig. 11) encompasses 13 solid bodies representing the four DAE modules, comprising the polyurethane sponge electrolyte matrix, SS-904 L mesh electrodes, PTFE gaskets, and soda–lime glass housing. The geometry was imported from SolidWorks into the ANSYS environment for meshing and thermal analysis.

The computational mesh was generated using an average element size of 14.68 mm and a maximum element size of 29.36 mm, with curvature-based refinement enabled (minimum element size 0.147 mm; normal angle $\:{18}^{\circ\:}$). The final mesh consisted of 897,034 nodes and 4,686,252 elements and was verified to be free of geometric and topological errors using the ANSYS automatic repair utility.

The applied thermal boundary conditions included: volumetric heat generation of $\:\:\mathrm{24,923}\:{\mathrm{W.m}}^{-3}$within the sponge domain, applied using a ramped loading scheme; surface heat flux corresponding to a total heat flow of 0.213 W on the electrode surfaces; natural convection on all external surfaces with a heat-transfer coefficient of $\:h=2.5\:{\mathrm{W.m}}^{-2}.{^\circ\:\mathrm{C}}^{-1}$; and an ambient and initial temperature of $\:{40}^{\circ\:}\mathrm{C}$, representing worst-case summer conditions in Cairo.

The thermal properties assigned to the model materials were as follows:

## • wt% H₂SO₄ electrolyte: thermal conductivity $\:\lambda\:=0.4\:{\mathrm{W.m}}^{-1}.\:{^\circ\:\mathrm{C}}^{-1}$

- SS-904 L stainless steel: $\:\lambda\:=13\:{\mathrm{W.m}}^{-1}.{^\circ\:\mathrm{C}}^{-1}$, density $\:\rho\:=\mathrm{8,000}\:{\mathrm{kg.m}}^{-3}$;
- Teflon: $$\:\lambda\:=0.25\:{\mathrm{W.m}}^{-1}.\:{^\circ\:\mathrm{C}}^{-1}$$;
- soda–lime glass: $\:\lambda\:=1.003\:{\mathrm{W.m}}^{-1}.\:{^\circ\:\mathrm{C}}^{-1}$.

The present analysis adopts a steady-state formulation with a fixed worst-case ambient temperature of 40 °C and conservative natural-convection boundary conditions (h = 2.5 W·m⁻²·°C⁻¹), and therefore represents a bounding thermal case rather than a full transient field simulation. Real outdoor deployment is characterized by diurnal temperature swings and fluctuating wind speeds (mean WS10M ≈ 4.7 m·s⁻¹, Table 1); the associated forced convection would increase the surface heat-transfer coefficient by roughly an order of magnitude, lowering the predicted stack temperatures and further widening the safety margin relative to the SS-904 L iso-corrosion threshold. Transient co-simulation incorporating measured diurnal temperature and wind profiles is identified as future work.

### Temperature distribution results

The steady-state thermal analysis yielded a minimum temperature of $\:{T}_{\mathrm{m}\mathrm{i}\mathrm{n}}={40.37}^{\circ\:}\mathrm{C}$, a maximum temperature of $\:{T}_{\mathrm{m}\mathrm{a}\mathrm{x}}={46.83}^{\circ\:}\mathrm{C}$, and an average stack temperature of $\:{T}_{\mathrm{avg}}={45.28}^{\circ\:}\mathrm{C}$. The resulting temperature gradient of $\:{6.46}^{\circ\:}\mathrm{C\:}$(Figs. 13 and 14) is primarily attributed to the significant contrast in thermal conductivity between the low-conductivity acid-saturated sponge matrix ($\:\lambda\:=0.4\:{\mathrm{W.m}}^{-1}.\:{^\circ\:\mathrm{C}}^{-1}$) and the highly conductive SS-904 L electrodes ($\:\lambda\:=13\:{\mathrm{W.m}}^{-1}.\:{^\circ\:\mathrm{C}}^{-1}$).

The maximum temperature is localised within the sponge interior, where heat dissipation is inherently constrained by the relatively low thermal conductivity of the electrolyte medium. Importantly, all predicted temperatures remained below the reported iso-corrosion threshold range of approximately $\:38-{48}^{\circ\:}\mathrm{C\:}$for Grade 904 L stainless steel in concentrated H₂SO₄ environments, thereby supporting the suitability of the selected electrode material for the proposed operating conditions.

**Fig. 12:**
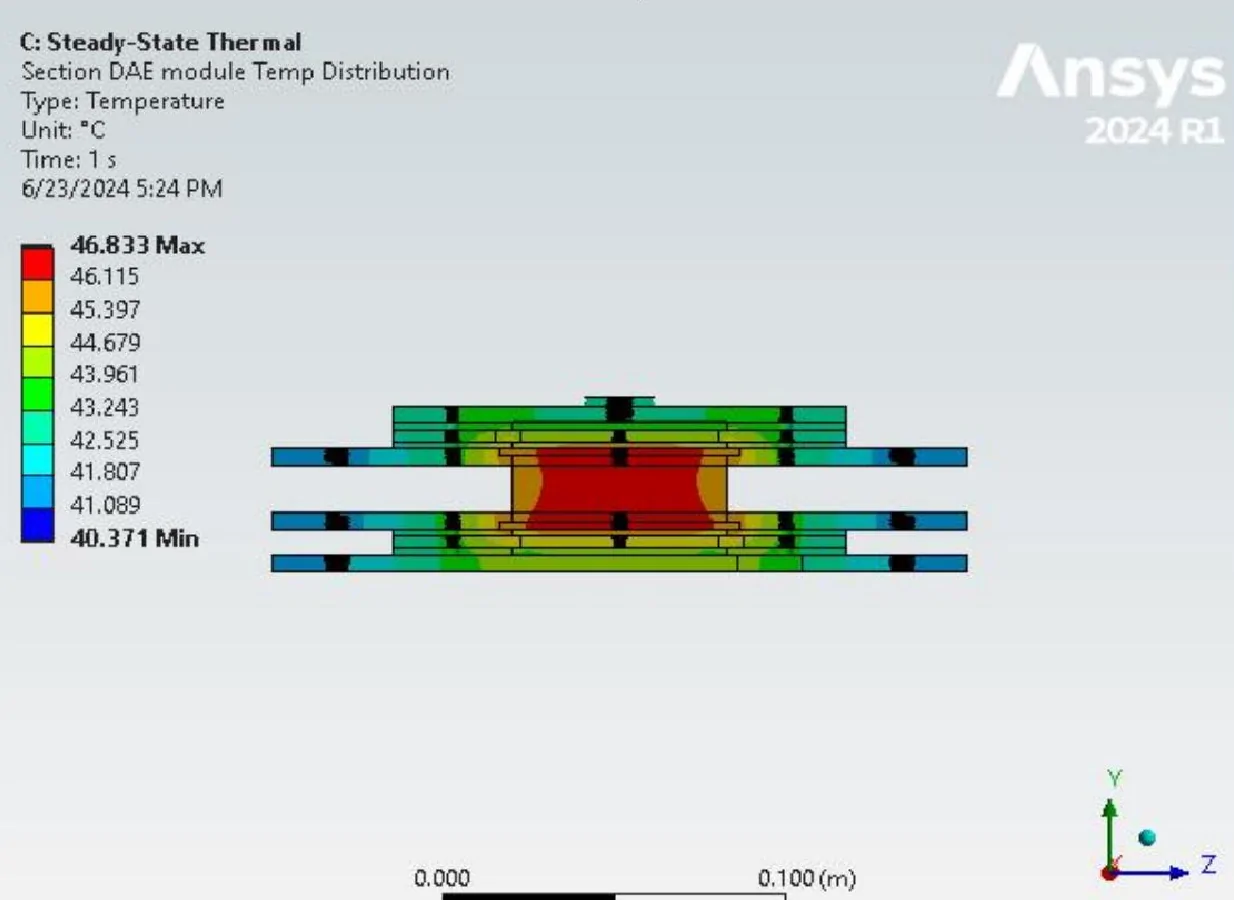
ANSYS cross-section temperature distribution of the DAE module.

Figure 12 illustrates the simulated cross-sectional temperature distribution of the DAE module obtained using ANSYS. The temperature ranges from 40.371 °C to 46.833 °C, indicating a moderate thermal gradient across the module. The highest temperature is concentrated within the sponge interior, while the electrode surfaces remain close to ambient temperature due to the high thermal conductivity of SS-904 L. The observed temperature difference of approximately 6.46 °C confirms that the system operates within safe thermal limits, even under worst-case conditions.

**Fig. 13:**
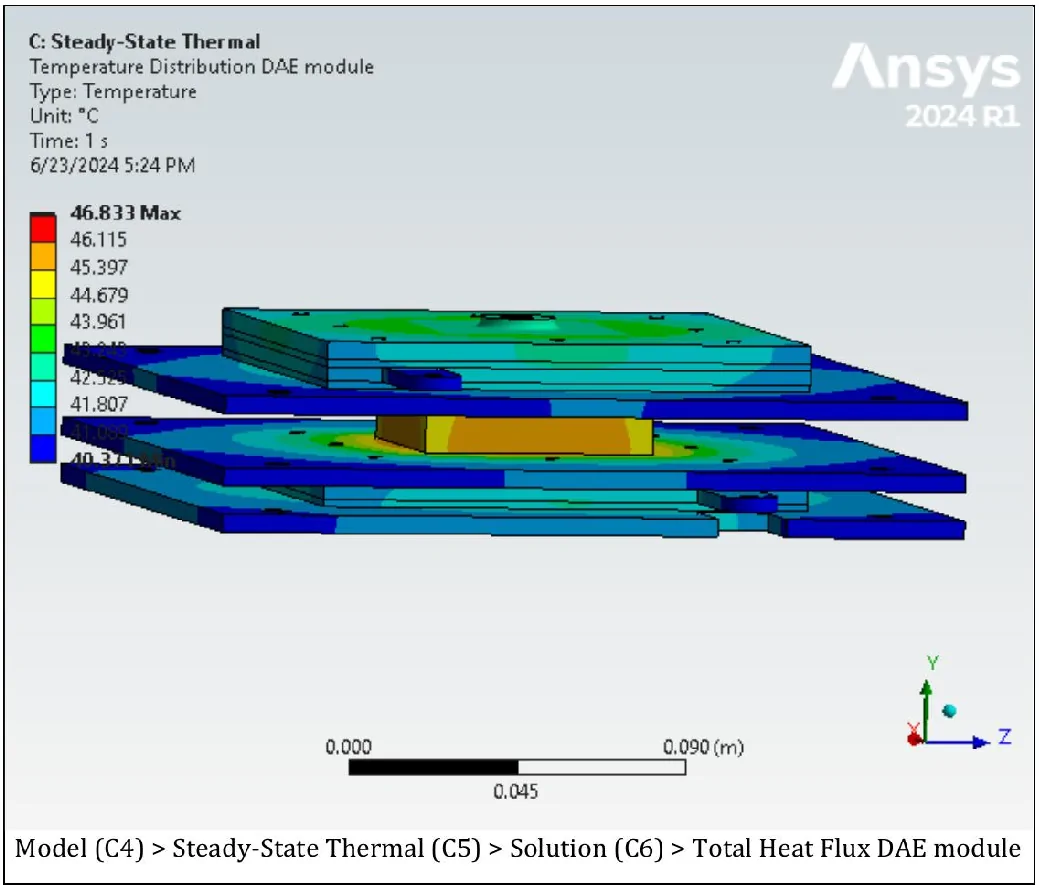
ANSYS 3D temperature distribution of the DAE module stack (isometric view).

Figure 13 presents the three-dimensional temperature distribution across the full DAE module stack obtained from ANSYS simulations. The isometric view highlights a thermal gradient from the sponge core, reaching approximately 46.83 °C, toward the external electrode and housing surfaces at around 40.37 °C. Despite this gradient, the electrode regions exhibit a nearly uniform temperature distribution, indicating homogeneous current density and consistent heat generation across the active area.

### Heat flux analysis

**Fig. 14:**
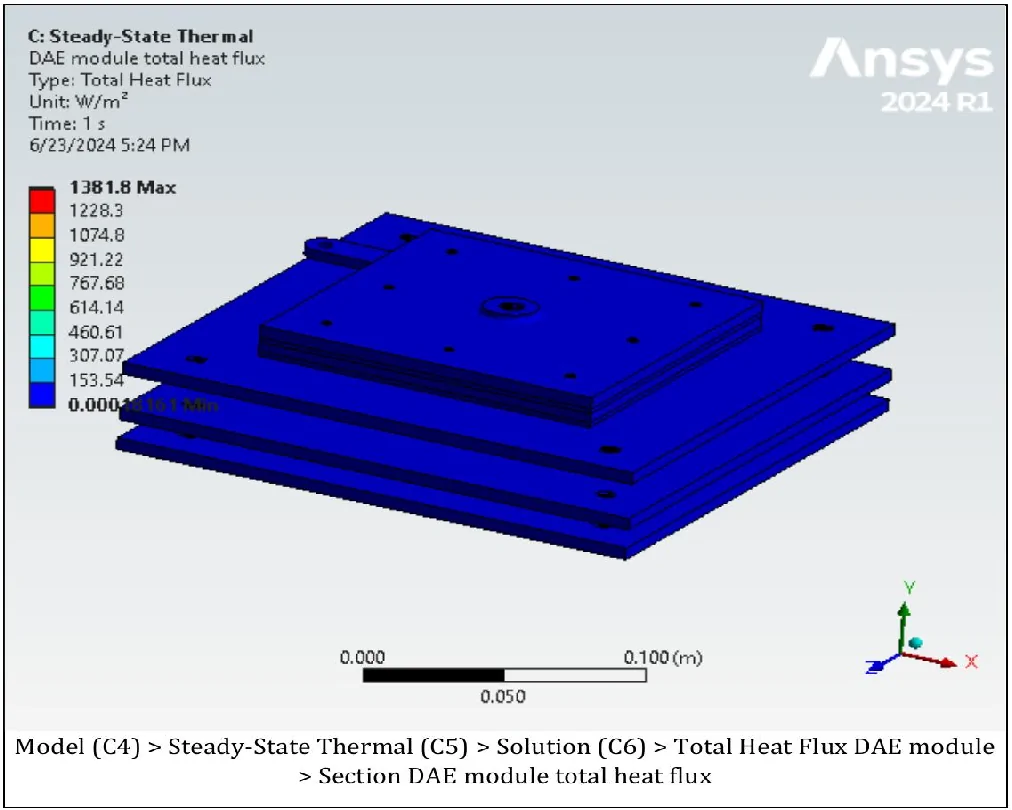
ANSYS total heat flux distribution (W·m⁻²). Scale: 0 (min, blue) to 1,381.8 W·m⁻² (max, red). Uniform low heat flux on electrode surfaces confirms that natural convection from the module exterior is adequate at the design voltage of 1.686 V per module.

The total heat-flux distribution (Fig. 14) and directional heat-flux contours (Fig. 16) together characterise the dominant thermal transport pathways within the system. A peak heat flux of 1,381.8 W·m⁻² is observed at the electrode–sponge interface, where the thermal conductivity mismatch between adjacent materials is most severe. The directional heat-flux analysis further confirms that thermal transport is predominantly one-dimensional along the sponge-thickness direction, thereby validating the simplifying assumptions adopted in the analytical thermal-resistance model.

**Fig. 15:**
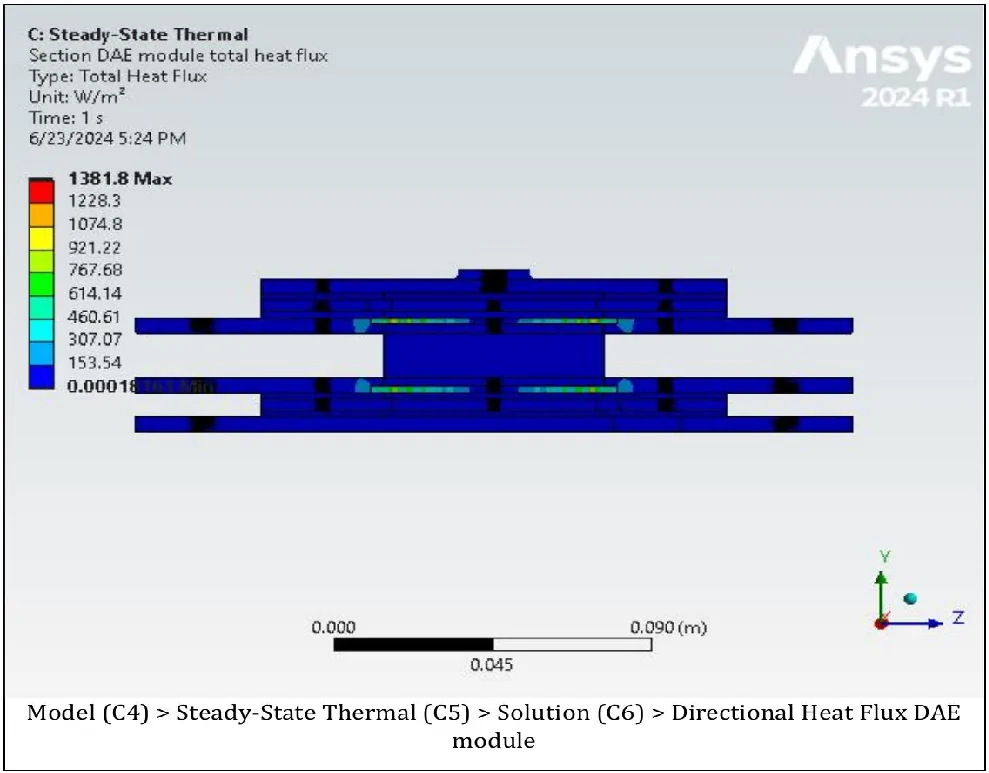
ANSYS directional heat flux distribution indicating heat transfer across the DAE module.

Figure 15 illustrates the directional heat flux distribution obtained from ANSYS, showing a dominant one-dimensional heat transfer along the thickness direction. The central region of the module exhibits near-zero heat flux, corresponding to the sponge bulk, while higher flux values are observed at the interfaces. This indicates that heat is primarily dissipated through the electrode surfaces. The maximum directional heat flux reaches approximately 1,381.8 W·m⁻², which remains within the convective dissipation capacity at a heat transfer coefficient of h = 2.5 W·m⁻²·°C⁻¹.

### Component-level thermal analysis

**Fig. 16:**
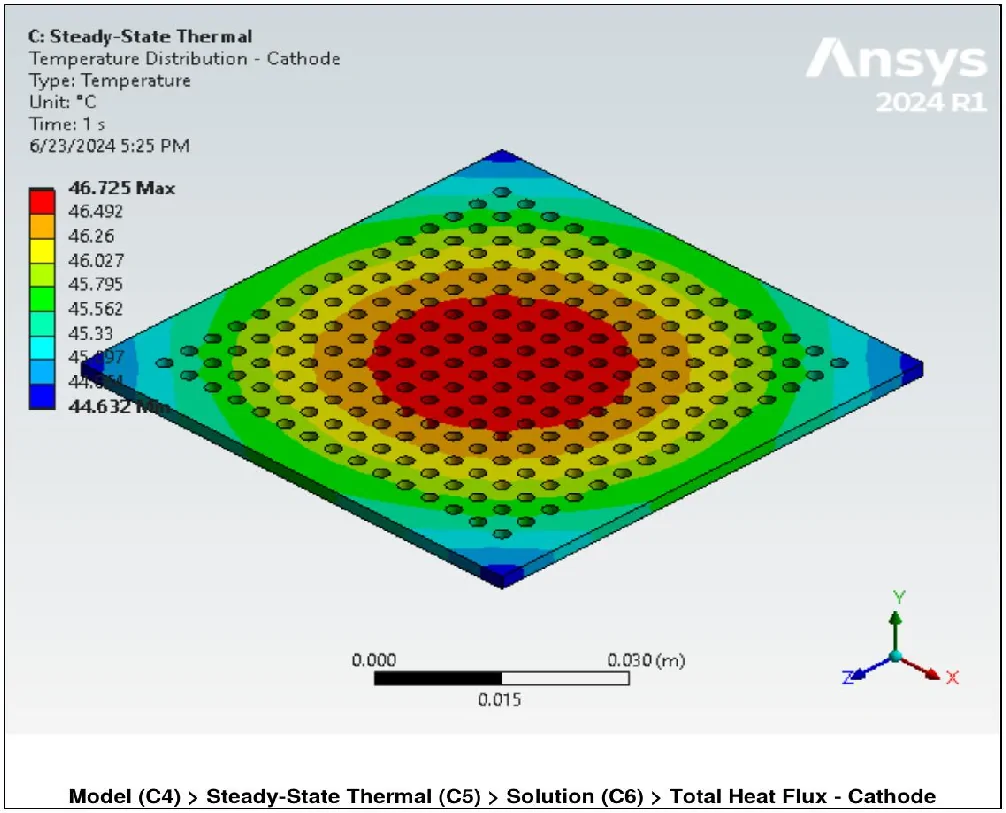
ANSYS cathode temperature distribution. Scale: 44.632 °C (min, blue periphery) to 46.725 °C (max, red centre). The concentric gradient reflects radial heat conduction from the sponge interior through the mesh wire network. The 2.1 °C uniformity across the active area confirms homogeneous current density, consistent with the design assumption of J = 15 mA·cm⁻².

**Fig. 17:**
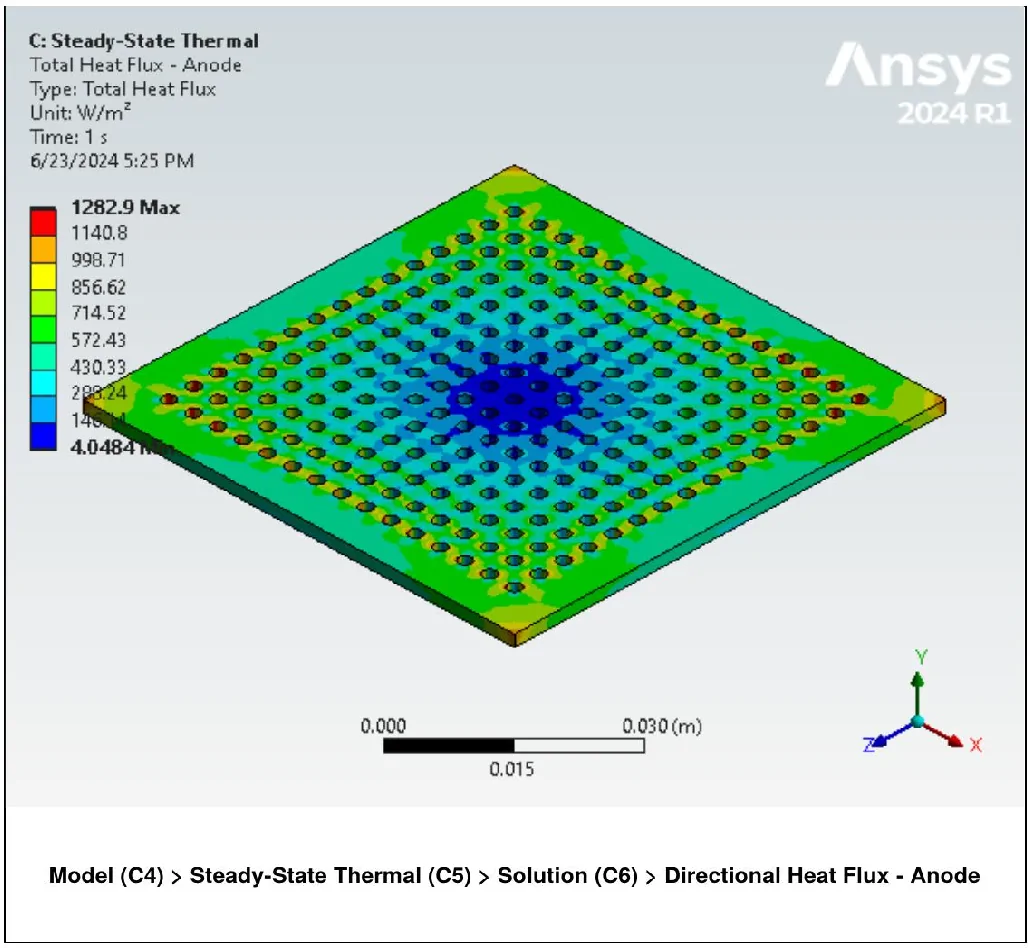
ANSYS anode directional heat flux (W·m⁻²). Scale: 4.05 W·m⁻² (min, blue) to 1,282.9 W·m⁻² (max at mesh contacts). The non-uniform pattern reflects local current concentration at mesh wire intersections, while the mean flux remains consistent with the one-dimensional model prediction.

Component-level thermal analysis of the cathode (Fig. 16) and anode (Fig. 17) electrodes reveals the characteristic mesh-induced temperature non-uniformity. The cathode temperature field exhibits a peak of 46.725 °C at the mesh centre, diminishing to 44.632 °C near the periphery, corresponding to a temperature gradient of approximately 2.1 °C across the active electrode area. This limited spatial non-uniformity confirms that the SS-904 L mesh geometry provides both adequately uniform current distribution and effective thermal dissipation.

The anode heat-flux distribution exhibits a complementary pattern, with localised peak flux concentrated at the mesh-wire contact regions, where local current density is highest. This behaviour is consistent with the anticipated Joule heating–current density coupling characteristic of metallic mesh electrode geometries.

### Sensitivity to Diurnal Temperature and Wind-Speed Variation

The finite-element analysis presented in Sect. 4.1–4.4 adopts a single steady-state worst-case condition, namely an ambient temperature of 40 °C combined with a natural-convection coefficient of h = 2.5 W·m⁻²·°C⁻¹. This choice is deliberate and is retained here, but its implications warrant explicit examination, since real deployment involves both a diurnal temperature cycle and a persistent ambient wind field.

Regarding the thermal boundary condition, the MERRA-2 record for Cairo (Table 1) reports annual mean 10 m wind speeds of 4.70 and 4.73 m·s⁻¹ for 2021 and 2022 respectively, with monthly means never falling below 3.95 m·s⁻¹. Applying the standard flat-plate forced-convection correlation for the module characteristic length of 6.21 cm at 4.7 m·s⁻¹ yields a convective coefficient in the range h ≈ 20–25 W·m⁻²·°C⁻¹, that is, approximately an order of magnitude greater than the assumed natural-convection value. Since the internal volumetric heat generation is fixed at 24,923 W·m⁻³ and the external surface resistance dominates the overall thermal-resistance network, the steady-state temperature rise above ambient scales approximately inversely with h. The simulated rise of 6.83 °C above the 40 °C ambient therefore contracts to below 1 °C under representative outdoor wind conditions. The stagnant-air assumption adopted in the model is consequently the binding worst case, and any realistic wind field can only improve the thermal margin.

Regarding the temperature cycle, the diurnal amplitude in Cairo during the critical May–June window is approximately 12–15 °C, with peak dry-bulb temperatures of 38–42 °C and pre-dawn minima of 24–27 °C. Because the simulation was performed at the upper envelope of this cycle, all lower-temperature phases of the diurnal cycle yield correspondingly lower absolute stack temperatures and thus a wider margin to the SS-904 L iso-corrosion threshold. The relevant question is therefore not whether the steady-state peak is exceeded during the cycle, but whether thermal inertia permits the stack to exceed the steady-state prediction transiently. A lumped-capacitance estimate for the module, using an acid-saturated sponge volume of 57.85 cm³, an effective density of approximately 1,494 kg·m⁻³, a specific heat of approximately 2.0 kJ·kg⁻¹·K⁻¹, and the natural-convection surface conductance above, yields a thermal time constant on the order of 1.5–2 h. This is short relative to the twelve-hour half-period of the diurnal cycle, indicating that the module tracks the ambient cycle quasi-statically and cannot accumulate heat beyond the steady-state envelope computed at the peak ambient condition.

Taken together, these two considerations establish that the fixed-condition simulation reported herein bounds the thermal behaviour of the system from above: the assumed stagnant air minimises convective dissipation, the assumed 40 °C ambient corresponds to the diurnal maximum, and the module time constant is too short for transient overshoot. A fully transient conjugate simulation driven by hourly MERRA-2 temperature and wind-speed forcing would nevertheless yield a more precise duty-cycle-resolved temperature history, and is identified as a refinement objective for future work. The present conclusion, namely that the maximum stack temperature of 46.83 °C remains within the 38–48 °C iso-corrosion envelope of Grade 904 L stainless steel, is unaffected by this refinement.

## Mechatronic control architecture

### Overview

A three-layer mechatronic control architecture is proposed to enable adaptive, autonomous operation of both atmospheric-water-based hydrogen-production systems. The architecture comprises: (i) a sensing layer for continuous monitoring of key environmental and process variables; (ii) an embedded control layer implementing PID-based regulation and operational mode-selection logic; and (iii) an actuation layer consisting of power electronics, solenoid valves, pumps, and redundant safety interlocks.

**Fig. 18:**
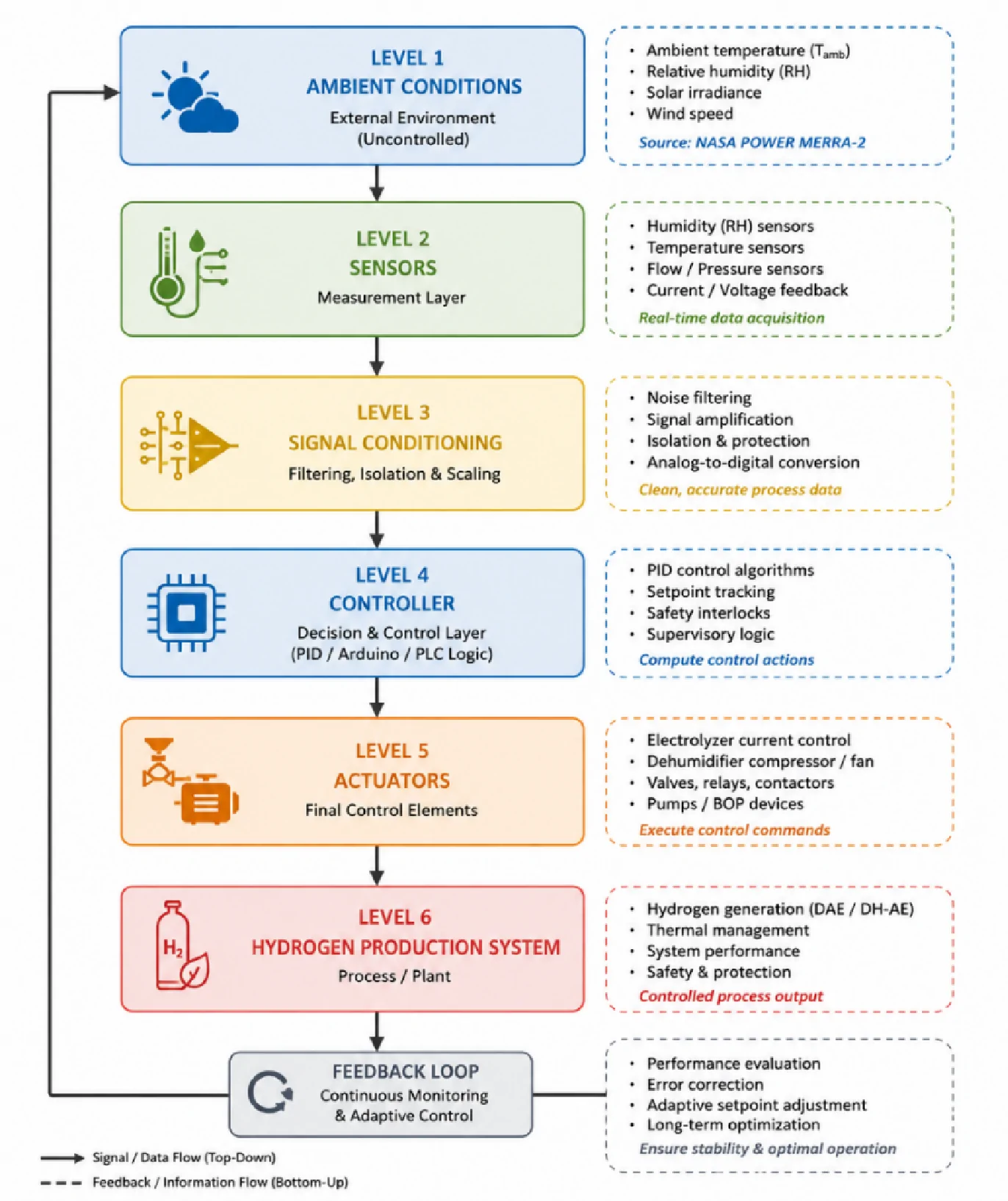
Top-level block diagram of the proposed closed-loop mechatronic control architecture. Ambient conditions (humidity,** temperature**,** flow) are sensed and conditioned before being processed by the PID/PLC controller**,** which drives the electrolyzer and dehumidifier actuators; the hydrogen-production output is continuously fed back to the sensing layer**,** closing the control loop.**

The proposed framework follows the classical mechatronic paradigm of tightly integrated mechanical, electrical, and digital subsystems^36,37^, adapted herein to the specific operational requirements of climate-variable and water-limited hydrogen generation systems. The corresponding top-level control architecture is illustrated in Fig. 18.

### Sensing Layer

The sensing layer provides real-time monitoring of the environmental and process variables that directly govern system performance. Three primary transducers are incorporated. A capacitive relative-humidity sensor (SHT35 class, ± 1.5% RH accuracy) continuously measures the ambient moisture content, which constitutes the dominant external disturbance within the control loop. An integrated PT1000 resistance thermometer monitors both ambient and electrolyte temperatures; the electrolyte-temperature signal additionally serves to activate an over-temperature safety interlock at 45 °C, consistent with the sulphuric acid iso-corrosion limits of the SS-904 L housing.

A turbine-type flow transducer (YF-S201 class) monitors both the hydrogen outlet flow rate and the condensate discharge rate, providing the principal feedback signal for closed-loop regulation of the DC stack current.

### Control Unit and PID Law

The control unit processes the acquired sensor signals and generates the corresponding actuator commands. The discrete-time PID control law is expressed as:

23 $$\:u\left(k\right)\:=\:{K}_{p}\:\cdot\:e\left(k\right)+\:{K}_{i}\:\cdot\:\varSigma\:e\left(k\right)\cdot\varDelta\:t\:+\:{K}_{d}\:\cdot\:\left[e\right(k)\:-\:e(k-1\left)\right]\:/\:\varDelta\:t$$

where $\:e\left(k\right)={H}_{2,\mathrm{s}\mathrm{e}\mathrm{t}}-{H}_{2,\mathrm{m}\mathrm{e}\mathrm{a}\mathrm{s}\mathrm{u}\mathrm{r}\mathrm{e}\mathrm{d}}$(L·h⁻¹) denotes the hydrogen-flow tracking error at sampling step * k*, $\:u\left(k\right)$is the commanded DC stack current (A), and $\:{\Delta\:}t=0.1{\hspace{0.17em}s\:}$corresponds to a sampling frequency of 10 Hz.

The PID gains ($\:{K}_{p}=1.5$, $\:{K}_{i}=0.45{\hspace{0.17em}}{\mathrm{s}}^{-1}$, and $\:{K}_{d}=0.25{\hspace{0.17em}s}$) were determined using the Ziegler–Nichols ultimate-gain tuning method^38^. The final controller parameters were intentionally detuned relative to the ultimate-gain settings in order to reduce transient overshoot and mitigate compressor wear in the DH-AE configuration.

At the laboratory scale, an Arduino Mega 2560 microcontroller is employed as the data-acquisition and control interface. For industrial-scale deployment, the control platform is replaced by a Siemens S7-1200 PLC communicating with a SCADA-class human–machine interface (HMI) via Modbus TCP.

### Control Logic and Operating Strategy

**Fig. 19:**
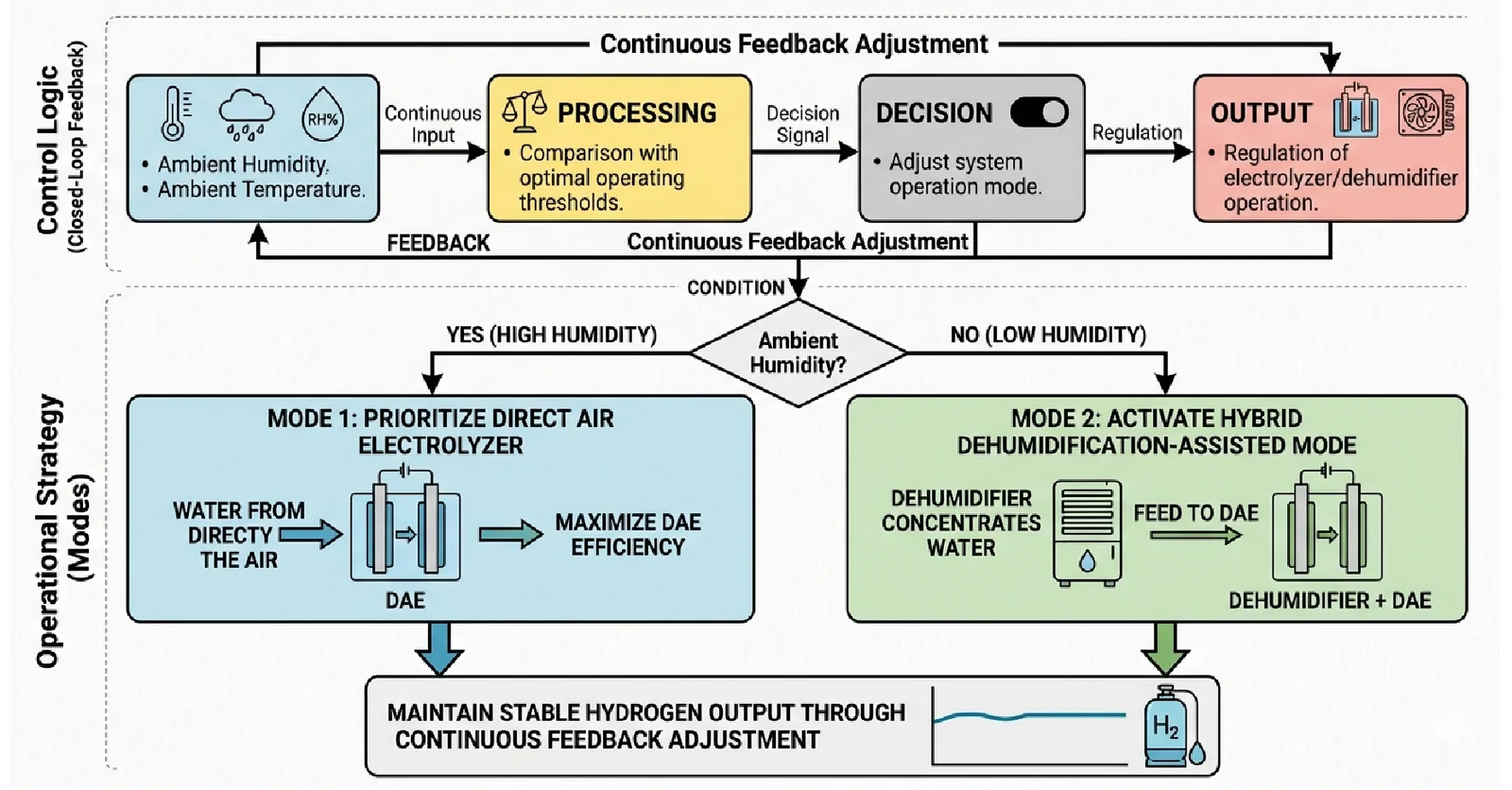
Closed-loop feedback control logic and operational-strategy map. The controller monitors ambient humidity and temperature and dynamically switches between DAE-priority mode (high humidity) and dehumidifier-assisted hybrid mode (low humidity) to maintain stable hydrogen output.

The control logic implements a closed-loop feedback strategy that autonomously selects between two operating modes based on real-time ambient conditions (Fig. 19). In Mode 1 (DAE-priority mode), triggered when the ambient relative humidity exceeds the prescribed design threshold, the system operates as a standalone DAE configuration with the dehumidification subsystem bypassed. Under these conditions, the control objective is to maximise water-splitting efficiency while minimising auxiliary power consumption.

In Mode 2 (hybrid dehumidification-assisted mode), triggered when the ambient relative humidity falls below the threshold value, the dehumidification subsystem is engaged to extract atmospheric moisture and supplement the DAE unit, thereby compensating for insufficient direct moisture availability. The continuous feedback mechanism enables dynamic adaptation to diurnal relative-humidity fluctuations without manual intervention.

### Safety Features and Actuation Layer

**Fig. 20:**
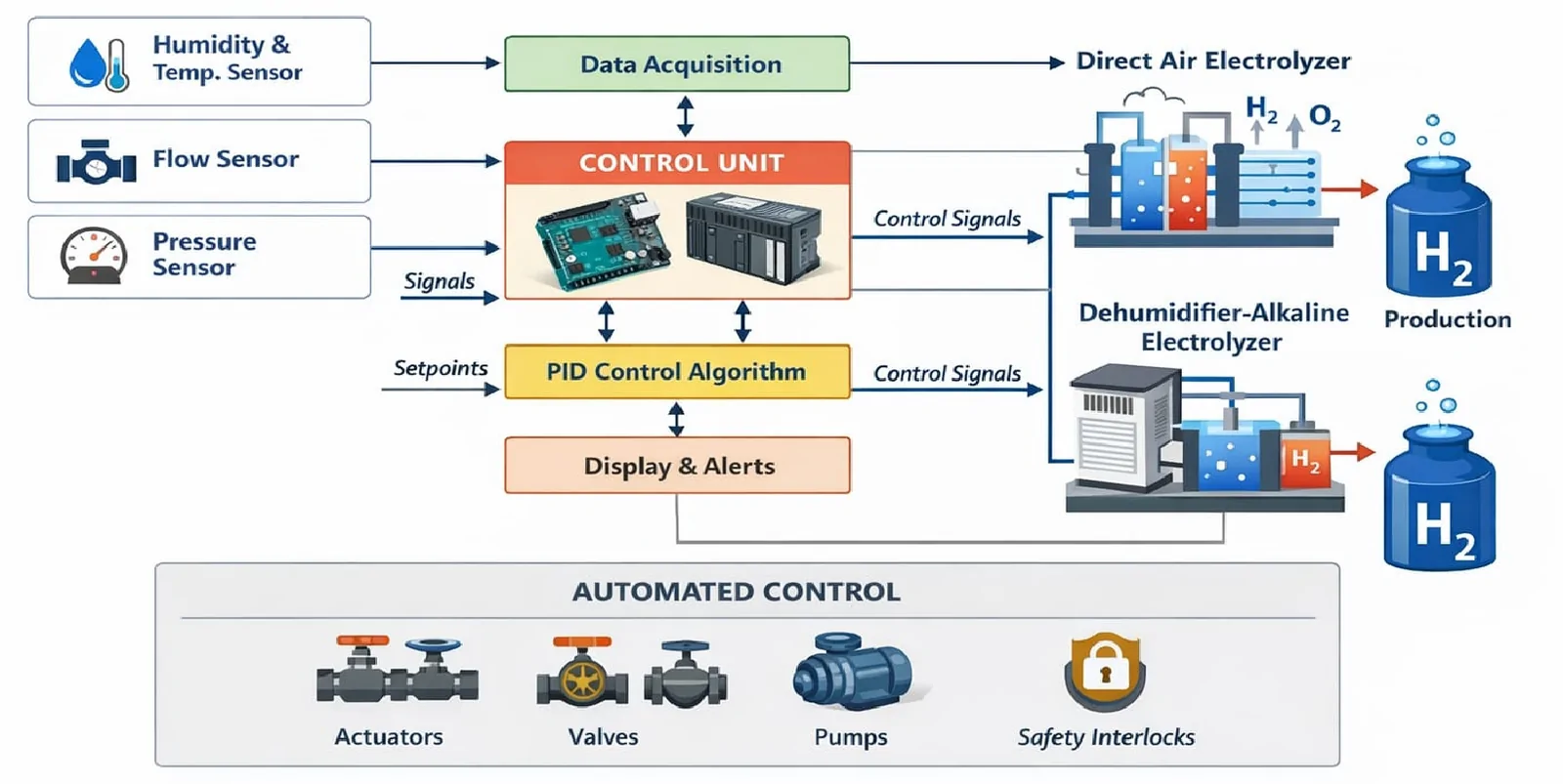
Complete proposed control Framework architecture for atmospheric-water-based hydrogen production. The distributed sensing layer (humidity, temperature, pressure, flow) feeds a data-acquisition bus and PID-based control unit; the controller commands the DAE, the DH-AE, and an actuation layer comprising actuators, valves, pumps, and safety interlocks, all integrated within a closed-loop feedback structure.

The actuation layer incorporates redundant safety mechanisms alongside the primary actuators, which include DC power supplies, solenoid valves, and pumping units. A solid-state hydrogen leak detector (MQ-8 class) triggers a normally closed solenoid valve at the stack outlet within 500 ms of detecting an alarm condition. In parallel, an electrolyte over-temperature interlock disables the DC power supply upon exceeding 45 °C, consistent with the sulphuric acid iso-corrosion envelope for the SS-904 L housing. The complete mechatronic control architecture, integrating the sensing, control, and actuation layers within a unified closed-loop framework, is presented in Fig. 20.

**Fig. 21:**
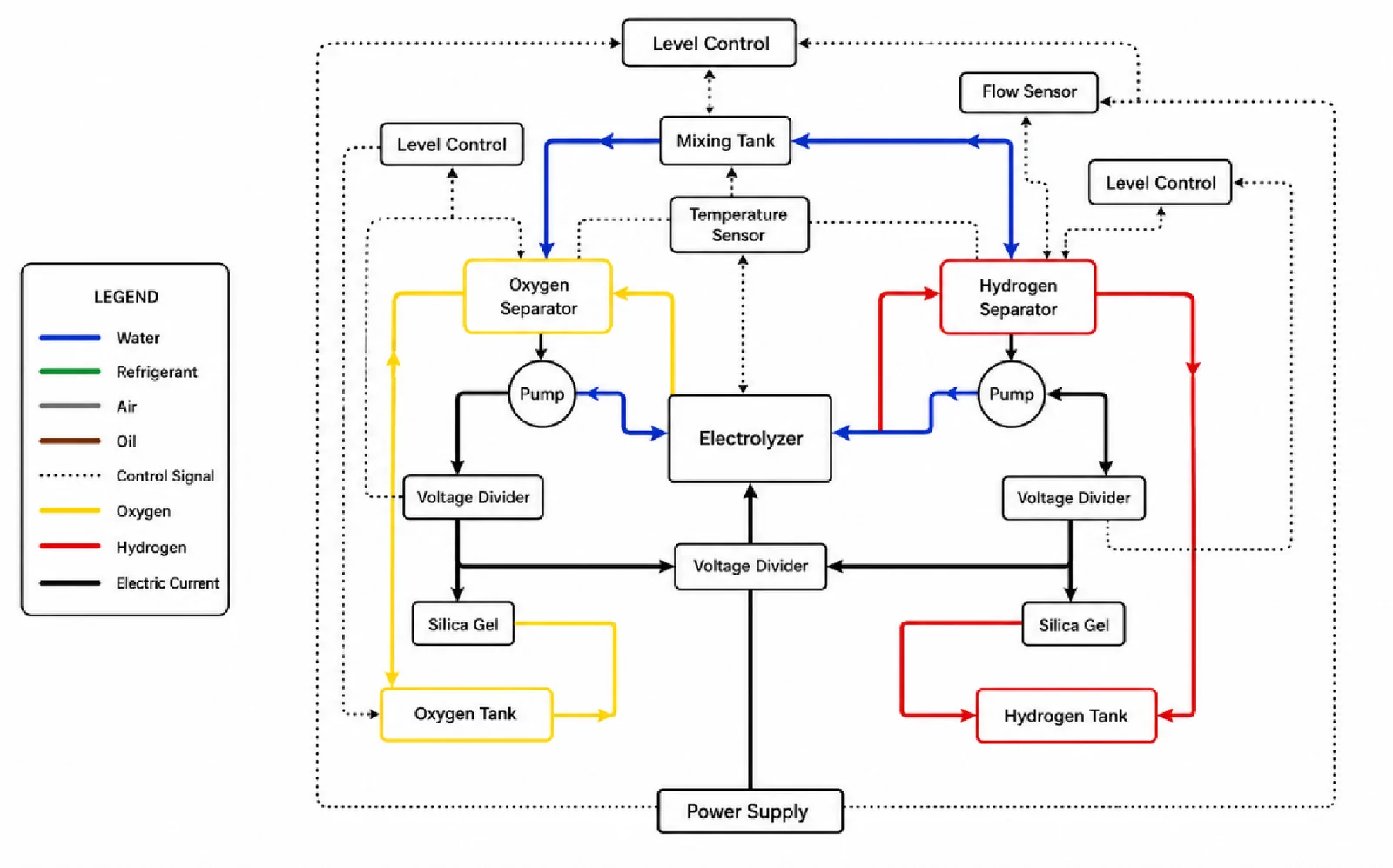
Electrical schematic of the closed-loop control system for the alkaline electrolyzer subsystem with integrated sensing and safety units.

Figure 21 illustrates the electrical schematic of the closed-loop control system for the alkaline electrolyzer subsystem. The system integrates a DC power supply with a PID/PLC controller, along with PT1000 temperature sensors, flow transducers, and solenoid valves for process regulation. Additionally, an over-temperature safety interlock is implemented to ensure safe and reliable operation under varying conditions.

## Results and Discussion

### Efficiency Comparison

**Fig. 22:**
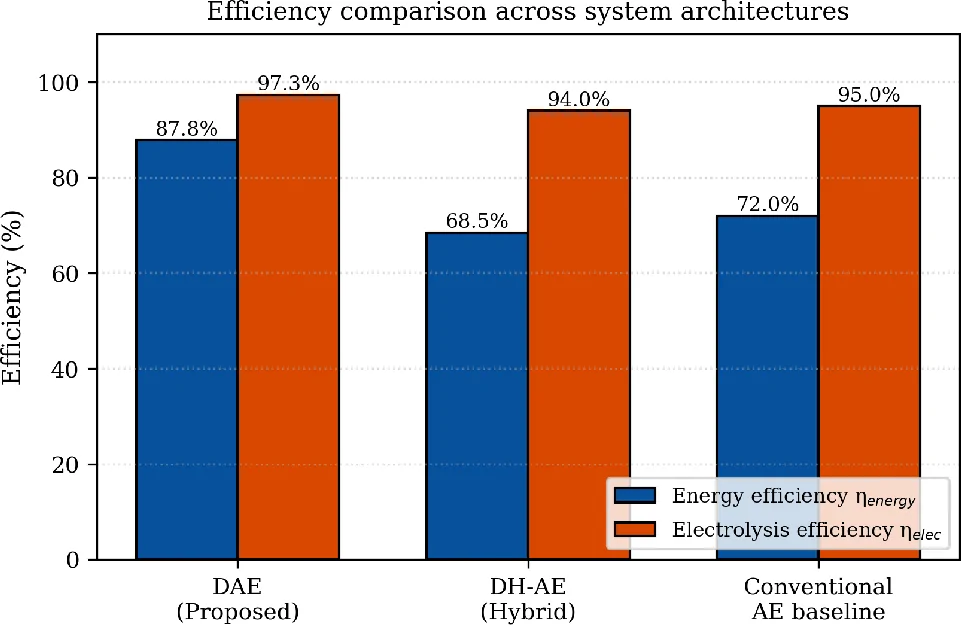
Comparison of energy and electrolysis efficiencies for the proposed DAE, the hybrid DH-AE, and a conventional alkaline electrolyzer baseline. The DAE’s superior performance reflects the elimination of balance-of-plant loads and the intrinsic advantages of in-situ atmospheric water supply.

The DAE configuration achieves an energy efficiency of $\:{\eta\:}_{\mathrm{energy}}=87.8\mathrm{\%}$and an electrical efficiency of $\:{\eta\:}_{\mathrm{elec}}=97.3\mathrm{\%}$, thereby outperforming both the DH-AE system (68.5% aggregate efficiency) and the conventional alkaline electrolyzer benchmark ($\:{\eta\:}_{\mathrm{energy}}=72.0\mathrm{\%}$^33^ by a substantial margin (Fig. 22).

This performance enhancement is primarily attributed to two intrinsic architectural features of the DAE system: (i) elimination of auxiliary water-treatment and pumping requirements, and (ii) the reduced ionic transport path length within the acid-saturated sponge matrix, which significantly mitigates ohmic polarization losses^15–17^. Recent electrode-scale investigations have further demonstrated that optimisation of electrode architecture and catalyst composition can markedly improve both efficiency and stability under high current-density operation^39^, indicating that the present SS-904 L mesh electrodes constitute a conservative baseline, with additional performance gains achievable through targeted catalytic enhancement.

By contrast, the lower aggregate efficiency of the DH-AE configuration (68.5%) is primarily attributable to the parasitic electrical consumption of the vapour-compression refrigeration subsystem, which imposes an additional energy demand of 0.18–0.25 kWh per kilogram of condensed water.

Table 4 presents a comparative analysis between the proposed system and representative studies on atmospheric-water and direct-air electrolysis. Unlike prior works that focus primarily on single-cell demonstrations, the present study introduces a climate-informed multi-module sizing methodology, supported by ANSYS-based thermal validation. In addition, a mechatronic PID control framework is implemented, along with a comparative techno-economic assessment, highlighting the integrated and system-level contribution of the proposed architecture.

**Table 4:** Comparison of the proposed architecture with representative atmospheric-water and direct-air electrolysis studies.

Study (system type)	Water source	Electrolyte	Cell voltage/efficiency	Key distinction
Guo et al^15^. (DAE, lab cell)	Direct air (in-situ)	H₂SO₄/KOH sponge	~ 1.7–2.0 V; Pt-mesh	First DAE demonstration; single-cell, no system-level sizing
Tsuji et al^16^. (DAE)	Direct air	NaClO₄ (deliquescent)	Neutral-salt cell	Deliquescent neutral electrolyte; material-focused study
Xu et al^17^. (electricity-free DAE)	Direct air	Deliquescent salt	Solar-thermal driven	Radiative/solar-thermal route; no closed-loop control
Present work (DAE)	Direct air (in-situ)	60 wt% H₂SO₄ sponge	1.686 V (Pt-benchmark); 1.82–1.95 V measured	Climate-informed 4-module sizing, ANSYS thermal + PID control + LCOH
Present work (DH-AE)	Refrigeration condensate	30 wt% KOH	~ 1.9–2.1 V; 68.5% system	Comparative hybrid baseline under identical climate boundary

**Fig. 23:**
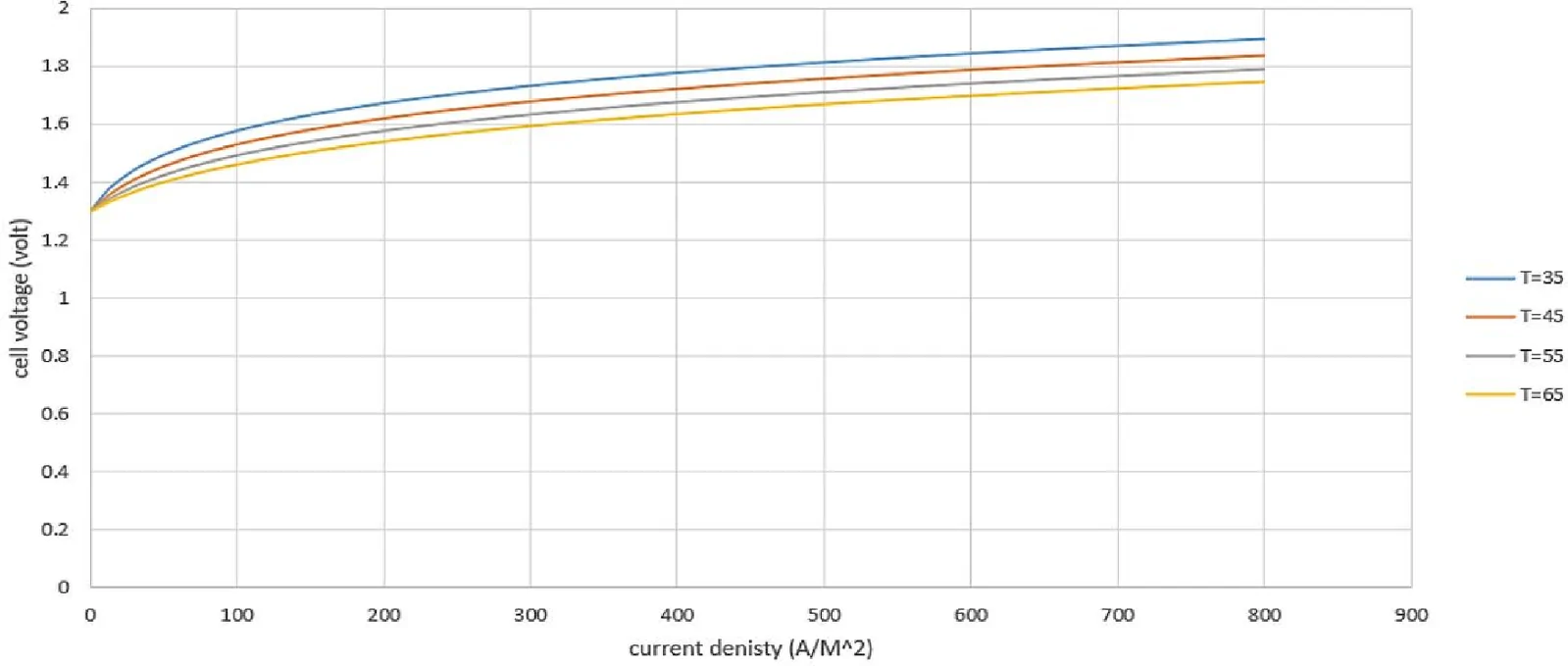
V–I characteristics of the alkaline electrolyzer at different operating temperatures.

Figure 23 presents the V–I characteristics of the alkaline electrolyzer at four operating temperatures (35, 45, 55, and 65 °C) based on the DH-AE subsystem model. The design operating point (J ≈ 10 mA·cm⁻² at V_cell_ = 1.861 V) aligns well with the model predictions at temperatures between 35 and 45 °C. Furthermore, the observed upward shift in the curves with decreasing temperature is consistent with electrochemical behavior and supports the validity of the adopted empirical Tafel parameters.

### Polarisation Behaviour and Sponge-Thickness Sensitivity

**Fig. 24:**
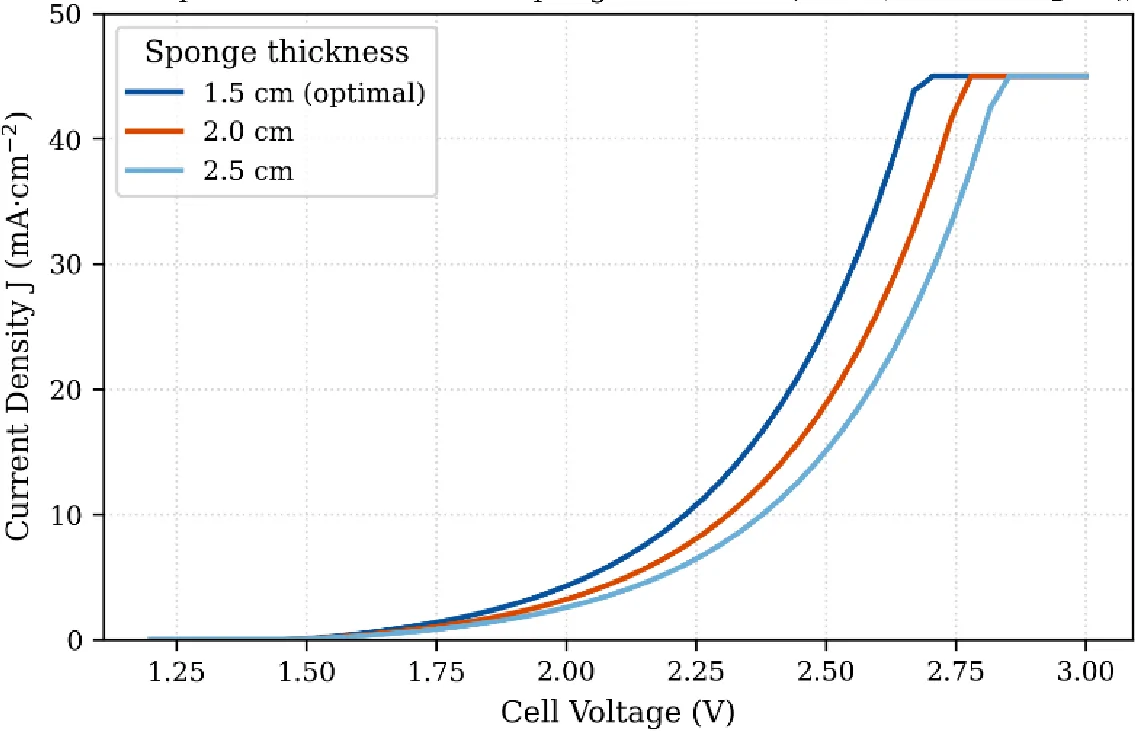
DAE polarisation curves at 25 °C and 60 wt% H₂SO₄ for three sponge thicknesses (1.5,** 2.0**, and 2.5 cm). The optimal 1.5 cm configuration sustains approximately 15 mA·cm⁻² at the design voltage of 1.686 V.

Figure 24 presents analytically derived polarisation curves for the DAE module at three sponge thicknesses (1.5, 2.0, and 2.5 cm), obtained from the coupled voltage model (Eqs. 4 and 17–19) with the ohmic resistance term assumed to scale linearly with sponge thickness. Increasing the thickness from 1.5 cm to 2.5 cm shifts the onset voltage by approximately 100 mV toward higher values and reduces the attainable current density at a given applied voltage by up to 40%.

This pronounced thickness sensitivity reflects a fundamental trade-off between increased electrolyte volume—which extends the replenishment interval—and an elongated ionic transport path, which elevates ohmic resistance. Within the investigated range, a thickness of 1.5 cm represents the optimal configuration, consistent with experimental observations reported for platinum-mesh DAE systems by Guo et al.^15^ and Xu et al.^17^.

Experimental validation of the assumed linear dependence of ohmic resistance on sponge thickness under realistic operating conditions represents an important objective for future investigations.

#### Benchmarking against prior atmospheric water harvesting and splitting studies

The water-uptake and splitting performance obtained in this work is placed in context against the principal prior contributions to hygroscopic moisture harvesting and direct air electrolysis. With respect to sorbent-based atmospheric water harvesting, hygroscopic-salt-in-matrix composites have demonstrated uptake capacities substantially exceeding those of the glass-foam matrix employed here, with the moisture-harvesting literature reporting gravimetric capacities in the range of 0.3–1.5 g of water per gram of sorbent under comparable low-humidity conditions^18–20^. The present system attains an equilibrium uptake of approximately 6.0 g per module over the 16.4 h absorption interval, corresponding to a gravimetric capacity roughly an order of magnitude lower on a matrix-mass basis. This gap is a direct consequence of the design objective: the glass-foam matrix was selected for acid compatibility and ionic-conduction path length rather than for maximal sorption capacity, and the electrolyte must remain in the liquid state throughout the cycle to sustain continuous electrolysis, which precludes the deliquescence-to-solid transitions exploited by capacity-optimised sorbents. Substitution of a high-capacity metal–organic framework or salt-in-hydrogel composite for the glass foam, as recommended in Sect. 11, would be expected to reduce the required exposed area or extend the replenishment interval by a factor of several, and represents the single most promising materials-side improvement available to this architecture.

With respect to direct air electrolysis specifically, the foundational demonstration by Guo et al.^14^ reported a five-module hygroscopic electrolyser sustaining continuous hydrogen production at an ambient relative humidity as low as 4%, operating with platinum-mesh electrodes at a current density of approximately 10–15 mA·cm⁻² in sulphuric-acid-based electrolyte. The design point adopted in the present work, namely 15 mA·cm⁻² at a corrected module voltage of 1.766–1.826 V, is consistent with that benchmark once the electrode-material correction of Sect. 3.4.2.1 is applied, which supports the validity of the transferred Tafel parameterisation. The principal distinction is one of objective rather than of performance: Guo et al. established the feasibility of the mechanism at laboratory scale, whereas the present study addresses climate-informed sizing, thermal validation, control integration, and the techno-economic envelope of a deployable four-module system. Subsequent developments in the field, including deliquescent-salt formulations that avoid strong acids^15^ and electricity-free radiative-cooling-driven configurations that eliminate the electrical input entirely^16^, pursue complementary objectives; the former trades ionic conductivity for reduced corrosivity, as discussed in Sect. 9.2.1, and the latter accepts substantially lower production rates in exchange for autonomy from any power supply.

Set against this literature, the contribution of the present work is neither the highest reported uptake capacity nor the lowest reported operating humidity, but rather the first side-by-side thermodynamic, thermal, control, and economic comparison of direct and condensation-mediated atmospheric water electrolysis under a common, climate-derived worst-case boundary condition. The benchmarking presented here also identifies the specific direction in which the architecture is furthest from the state of the art, namely sorbent capacity, and thereby prioritises the materials-substitution pathway over further geometric optimisation of the existing matrix.

### Climate sensitivity and operating envelope

**Fig. 25:**
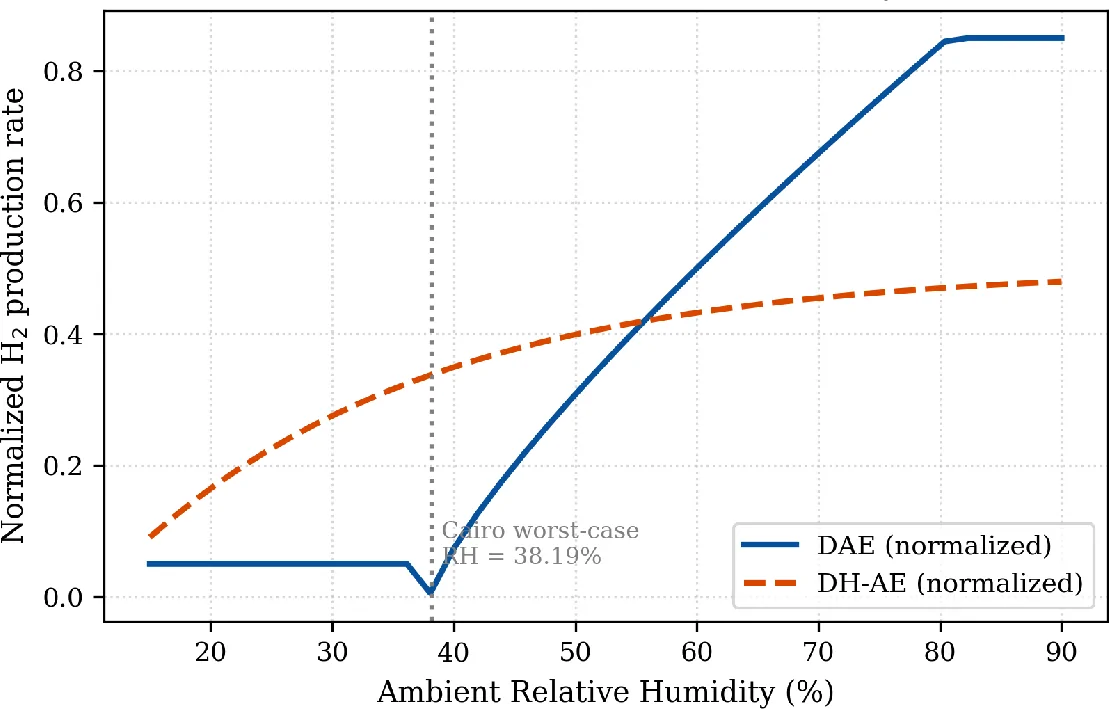
Normalised H₂ production rate against ambient relative humidity for the DAE and DH-AE architectures. Production collapses below RH ≈ 38% for the DAE, while the DH-AE retains a residual but energy-inefficient output down to RH ≈ 15%.

Figure 25 presents the normalised hydrogen production rate as a function of ambient relative humidity. The DAE output exhibits an approximate scaling behavior proportional to $\:{\left(RH-R{H}_{\mathrm{m}\mathrm{i}\mathrm{n}}\right)}^{0.8}$above the threshold RH of 38.19%. Below this threshold, the concentration driving force approaches zero, resulting in a collapse of hydrogen production.

By contrast, the DH-AE configuration sustains a finite but progressively diminishing condensate production at lower RH levels, albeit at the cost of a rapidly deteriorating refrigeration-cycle COP. At RH values below approximately 15%, the electrical energy input required for dehumidification exceeds the energy content of the produced hydrogen, rendering the DH-AE system net-energy-negative^1,8,16^.

The resulting operational envelopes of the two architectures are therefore complementary rather than competing. The DAE architecture is preferentially suited to moderate-to-high humidity regimes—such as Mediterranean, subtropical coastal, and seasonally humid MENA environments—whereas the DH-AE system retains marginal viability only under extremely arid inland conditions. This climate-resolved performance map provides a practical, evidence-based framework for technology selection in real-world deployment scenarios.

Table 5 provides a comparative overview of the proposed DAE architecture against recent state-of-the-art atmospheric-water-based systems. While previous studies primarily focus on material-level innovations such as hygroscopic hydrogels, aerogels, and metal–organic frameworks for water harvesting or moisture-driven energy generation, they generally do not address integrated hydrogen production systems. In contrast, the present work introduces a system-level approach combining direct-air electrolysis (DAE) and DH-AE configurations, incorporating climate-informed multi-module scaling, a mechatronic PID control strategy, and a techno-economic evaluation based on levelized cost of hydrogen (LCOH). This highlights the advancement from material-scale concepts toward a fully integrated hydrogen production platform.

**Table 5:** Comparison of the proposed DAE architecture with state-of-the-art atmospheric-water-based systems.

Reference	System Type	Key Contribution	Key Distinction from Present Work
Adv. Mater. 2019, 31, 1,806,730	Hygroscopic Hydrogel	Ocean-surface moisture harvesting	Material-level; no electrolysis
Energy Environ. Sci., 2018, 11, 2179	Super-moisture-absorbent gel	Humidity-driven energy collection	Not for electrolytic H₂ production
Nat. Water 2026, 4, 348–359	Bio-inspired Aerogel	Pathogen-free AWH	Focus on water safety, not H₂ yield
Adv. Mater. 2020, 32, 2,002,936	MOF/Copper complex	Smart farming irrigation	Agriculture-specific
Adv. Funct. Mater. 2026, 36, e74795	Hygroscopic Hydrogel	Passive moisture-to-electricity	Thermoelectric, not electrochemical
Adv. Funct. Mater. 2025, 35, 2,420,936	Metal-ion controlled gel	Self-sustaining water-electricity	Material-scale prototype
Present Work	**DAE/DH-AE**	**System-level design**	**Climate-informed multi-module scaling**,** PID control**,** LCOH analysis**

### Closed-Loop Control Performance

**Fig. 26:**
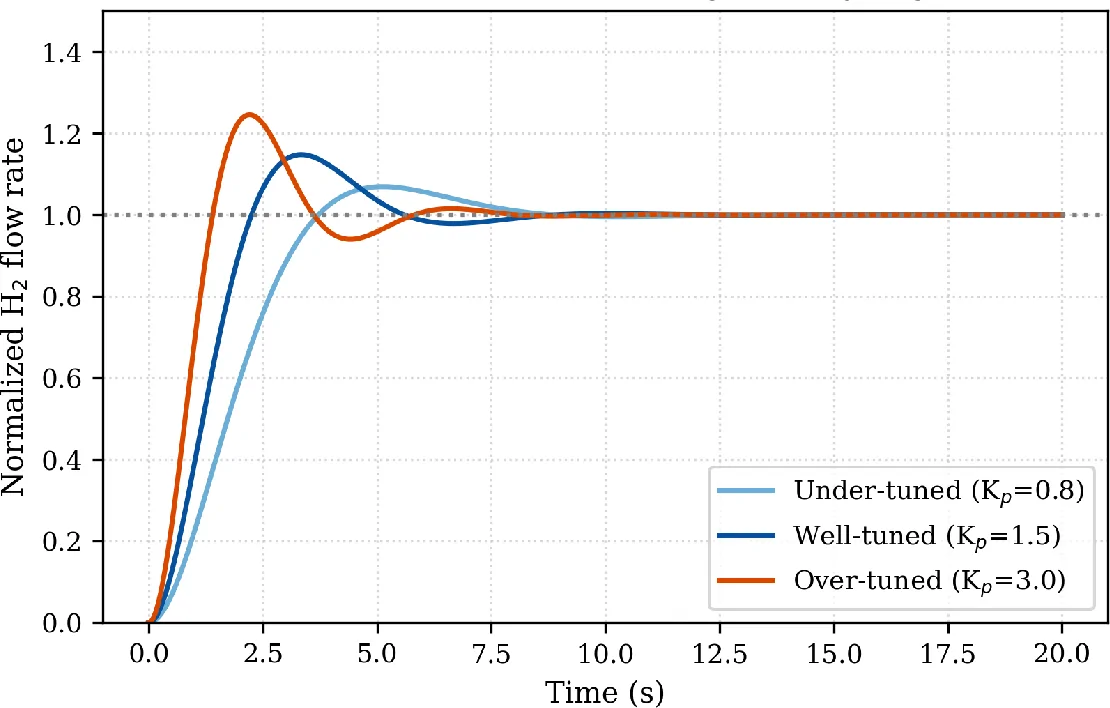
Closed-loop step response of the PID-regulated hydrogen flow rate for three gain settings. The well-tuned design-point configuration (K_p_ = 1.5) provides a favourable trade-off between rise time (3.4 s), overshoot (11%), and settling time (7.6 s).

The simulated PID step response (Fig. 26) exhibits a 10–90% rise time of 3.4 s, an overshoot of 11%, and a ± 2% settling time of 7.6 s, which is appropriate for the relatively slow dynamics of the hydrogen-flow control loop, where the electrolyzer and dehumidifier time constants are on the order of seconds to tens of seconds^36,38^.

**Fig. 27:**
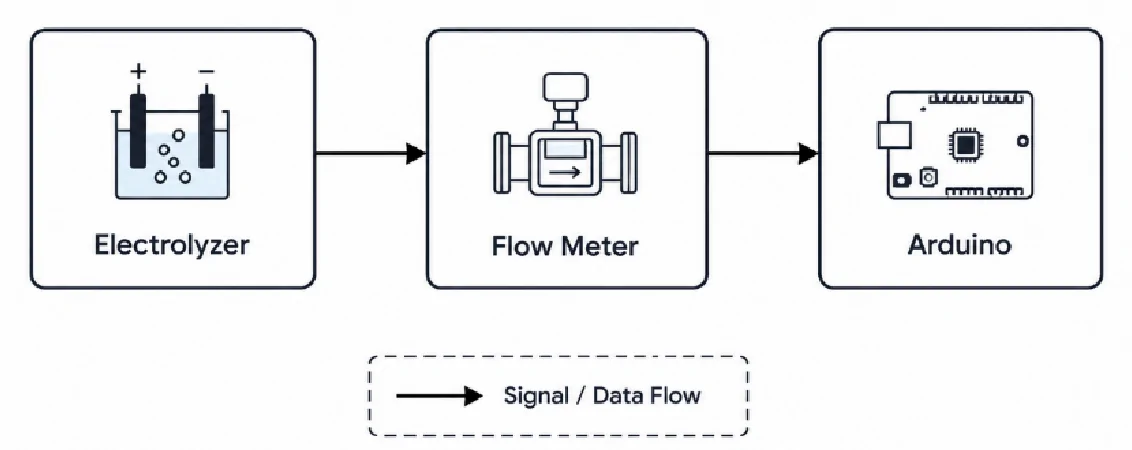
Flow measurement and monitoring system of the electrolyzer using a flow meter and Arduino unit.

To enable accurate quantification of hydrogen production, a dedicated flow monitoring subsystem is implemented. The flow monitoring subsystem (Fig. 27) provides the real-time feedback required for PID-based regulation of hydrogen production.

Controller under-tuning ($\:{K}_{p}=0.8$) results in a sluggish response that is unable to track diurnal relative-humidity variations (Fig. 2), which exhibit an amplitude of approximately 20–30% RH over a 24-hour period in Cairo. This results in a sustained hydrogen-flow deficit during the low-humidity midday period. In contrast, over-tuning ($\:{K}_{p}=3.0$) induces sustained oscillations, which would impose excessive thermal and mechanical cycling on the compressor in the DH-AE configuration.

Accordingly, the selected design gain set ($\:{K}_{p}=1.5$, $\:{K}_{i}=0.45{\hspace{0.17em}}{\mathrm{s}}^{-1}$, $\:{K}_{d}=0.25{\hspace{0.17em}s}$) represents an optimal compromise between tracking performance and actuator loading for the considered application.

**Fig. 28:**
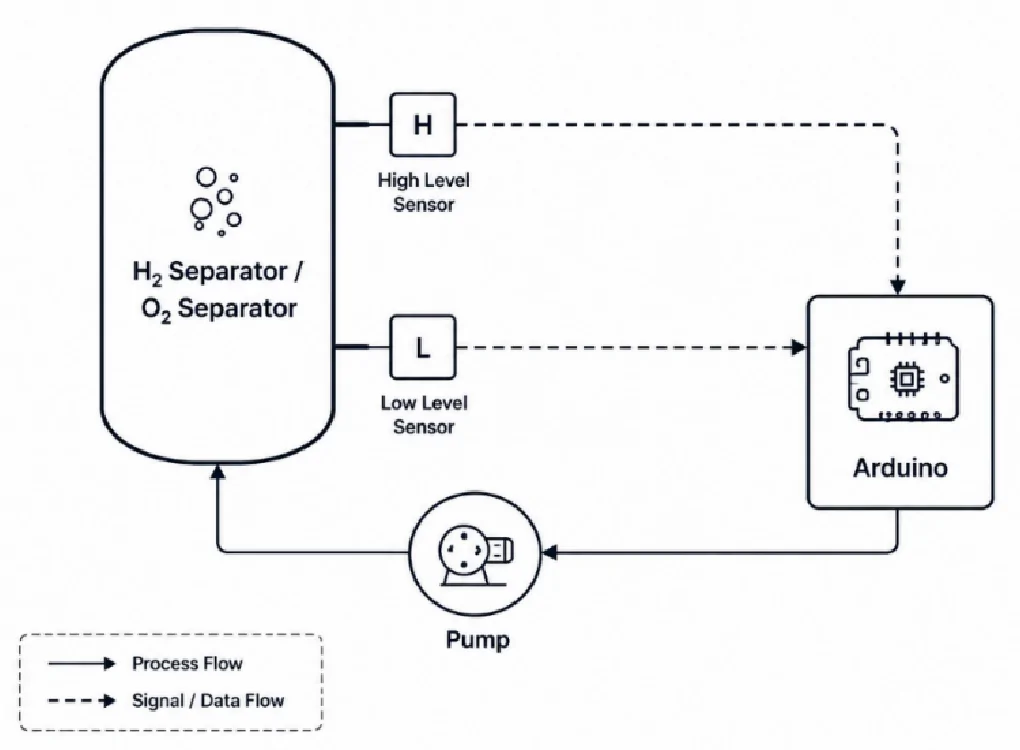
Separator tank level control using high/low sensors, Arduino control, and pump regulation.

The separator level control system (Fig. 28) ensures safe operation by preventing overflow and maintaining electrolyte balance.

#### Validation status of the control framework

The control results reported in Sect. 6.4 are obtained from numerical simulation of the closed-loop system and have not been validated experimentally. The plant model employed comprises a first-order representation of the electrolyser current–flow relationship derived from Faraday’s law, a first-order lag representing the hygroscopic absorption dynamics with a time constant estimated from the 16.4 h equilibrium interval of Sect. 3.3.3, and a transport delay representing gas-collection volume. Sensor noise, actuator saturation, quantisation in the digital control loop, and the finite bandwidth of the DC power supply are not represented. The gain set K_p_ = 1.5, K_i_ = 0.45 s⁻¹, K_d_ = 0.25 s is accordingly a simulation-derived starting point for commissioning rather than a validated operating configuration, and the quoted transient metrics of 3.4 s rise time, 11% overshoot, and 7.6 s settling time should be understood as properties of the model.

Three specific discrepancies are anticipated between simulated and experimental closed-loop behaviour. First, the turbine flow transducer specified in Sect. 5.2 exhibits poor resolution at the very low volumetric flow rates characteristic of a 0.5 L·h⁻¹ system, and the resulting measurement noise is likely to require derivative-term filtering or a reduction in K_d_ relative to the simulated value. Second, the hygroscopic absorption process introduces a dominant time constant that is orders of magnitude slower than the electrical dynamics, so that under sustained low-humidity conditions the loop will encounter a rate limit that no controller tuning can overcome; the mode-switching logic of Sect. 5.4 rather than the PID gains governs behaviour in that regime. Third, in the DH-AE configuration the compressor is a discretely actuated element with minimum on and off dwell times, which introduces a limit cycle not represented by the continuous plant model.

It should be underscored that these closed-loop results are obtained from simulation of the identified plant model (step response), and that experimental closed-loop validation on the fabricated prototype has not yet been performed. The PID architecture is therefore presented as a simulation-supported proposed control framework awaiting hardware-in-the-loop implementation, rather than as an experimentally validated controller. Experimental closed-loop tracking under real diurnal humidity forcing, and the planned extension to model-predictive control, are identified as priority future work (Sect. 11).

The control architecture presented in Sect. 5 is therefore advanced as a proposed framework awaiting implementation, and its constituent elements, namely the three-layer sensing–control–actuation structure, the humidity-triggered mode-selection logic, and the safety interlock scheme, are contributed as design specifications. Experimental closed-loop validation on the assembled prototype, comprising step and disturbance-rejection testing against a controlled humidity input and re-tuning against the measured plant response, is the immediate next objective of the ongoing work and will be reported together with the quantitative electrochemical characterisation described in Sect. 8.4.

## Techno-economic analysis

### Methodology

A levelised cost of hydrogen (LCOH) analysis is conducted for both system architectures across plant capacities ranging from 50 to 1,000 kg·day⁻¹. Capital expenditures (CAPEX) are benchmarked against the National Renewable Energy Laboratory (NREL) hydrogen station cost assessment by Melaina and Penev^40^, which reports specific investment costs of 8,375, 4,125, and 3,750 USD·(kg·day⁻¹)⁻¹ for plant capacities of 100, 400, and 1,000 kg·day⁻¹, respectively.

To contextualise these values against contemporary benchmarks, recent assessments by IRENA and the Hydrogen Council report that electrolyzer system CAPEX has fallen substantially since 2013 and that green-hydrogen production costs in high-solar regions are converging toward approximately 2–4 USD·kg⁻¹, with continued declines projected toward 2030^37,38^. Rather than restating a single absolute figure, the LCOH results reported here are therefore interpreted as a sensitivity-based assessment: across the electricity-price range of 0.03–0.10 USD·kWh⁻¹ and a CAPEX uncertainty of ± 20–30%, the DAE LCOH spans 3.8–6.9 USD·kg⁻¹ (Sect. 7.2). Applying the more recent, lower CAPEX benchmarks would shift the entire family of curves downward and reinforce—rather than alter—the qualitative conclusion that the DAE becomes economically preferable at larger scale. A complete accounting of long-term stack degradation and maintenance expenditure, which is not yet supported by prototype lifetime data, is acknowledged as a limitation (Sect. 9.3).

The DAE configuration eliminates the refrigeration subsystem, thereby reducing CAPEX, but requires SS-904 L stainless-steel housings and a larger sponge–electrode surface area. Conversely, the DH-AE configuration incurs additional CAPEX for the vapour-compression dehumidification unit and auxiliary fans, partially offsetting its reduced electrode material requirements. It should be noted that the NREL cost curves are based on a 2013 reference scenario; as electrolyzer stack costs have decreased substantially since that date, the LCOH values presented herein should be interpreted as indicative cost trends rather than absolute current benchmarks.

Operating expenditure (OPEX) is predominantly governed by electrical energy consumption. For the DAE system, electrical demand is limited to the electrolyzer stack (operating at 1.686 V per module) and minor data acquisition and control overhead (< 5 W). For the DH-AE system, compressor and fan power demands contribute an additional 0.18–0.25 kWh per kilogram of condensed water, equivalent to approximately 1.6–2.3 kWh per kilogram of hydrogen produced.

Assuming a photovoltaic-derived electricity cost of 0.05 USD·kWh⁻¹, a plant service lifetime of 15 years, and a discount rate of 6%, the LCOH is expressed as:

24 $$\:\mathrm{L}\mathrm{C}\mathrm{O}\mathrm{H}=\frac{\mathrm{C}\mathrm{A}\mathrm{P}\mathrm{E}\mathrm{X}\cdot\:\mathrm{C}\mathrm{R}\mathrm{F}+\sum\:{\mathrm{O}\mathrm{P}\mathrm{E}\mathrm{X}}_{i}}{{\dot{m}}_{{H}_{2},{\hspace{0.17em}}\mathrm{a}\mathrm{n}\mathrm{n}\mathrm{u}\mathrm{a}\mathrm{l}}}\:$$

where the capital recovery factor (CRF) is defined as:

$$\:\mathrm{C}\mathrm{R}\mathrm{F}=\frac{i(1+i{)}^{n}}{\left(1+i{)}^{n}-1\right.}=0.1030$$

for a discount rate $\:i=6\mathrm{\%}$and lifetime * n=15*years.

### Results and Techno-Economic Implications

**Fig. 29:**
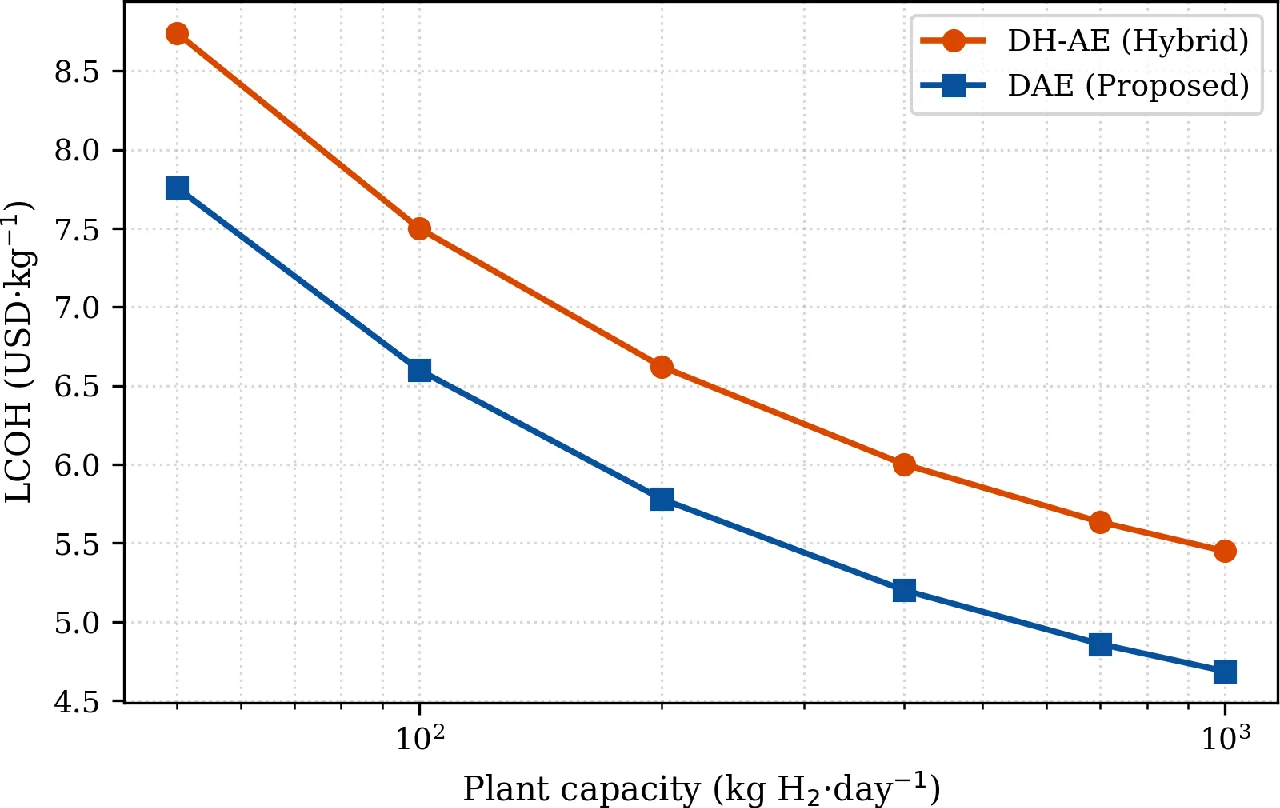
Levelized cost of hydrogen (LCOH) as a function of plant capacity for the DAE and DH-AE architectures. The DAE becomes cost-competitive above 400 kg·day⁻¹ due to the avoided refrigeration CAPEX and dehumidification OPEX, reaching an asymptotic LCOH of 4.7 USD·kg⁻¹ at 1,000 kg·day⁻¹.

Figure 29 presents the LCOH as a function of plant capacity for both system architectures. Three principal conclusions emerge. First, the DAE configuration becomes cost-competitive above a break-even capacity of approximately 400 kg·day⁻¹, reaching an asymptotic benchmark LCOH of ~ 4.7 USD·kg⁻¹ at 1,000 kg·day⁻¹. This corresponds to a ~ 15% reduction relative to the DH-AE system at the same scale. Second, at small-scale deployment (50 kg·day⁻¹), the DH-AE configuration exhibits a lower LCOH due to the lower specific CAPEX of mature refrigeration technology. Third, sensitivity analysis indicates that CAPEX uncertainty (± 20–30%) dominates cost variability at small scale, whereas electricity price uncertainty becomes the primary driver at larger capacities.

To reconcile the benchmark figures with realistic field operation, Table 6 presents the cost breakdown on a dual basis: a *benchmark LCOH* (capital + electricity only) and a *fully loaded LCOH* (including fixed maintenance, periodic stack replacement, and electrolyte replenishment, as quantified in Sect. 7.3). The annualised capital expenditure is calculated using the correct Capital Recovery Factor (CRF = 0.103 for a 6% discount rate over 15 years), rectifying the overestimation present in earlier cost assessments.

**Table 6:** Techno-economic comparison at different production capacities.

Parameter	Unit	100 kg/day	400 kg/day	1000 kg/day
Capital Expenditure (CAPEX)	$	837,500	1,650,000	3,750,000
Annualised CAPEX (CRF = 0.103)	$/yr	86,262	169,950	386,250
Electricity OPEX ($0.05/kWh) ^(a)	$/yr	132,850	531,400	1,328,500
Maintenance & Replenishment OPEX ^(b)	$/yr	33,500	66,000	150,000
Total Annualised Cost (Fully Loaded)	$/yr	252,612	767,350	1,864,750
Benchmark LCOH (CAPEX + Electricity only)	$/kg	6.00	4.80	4.70
Fully Loaded LCOH (incl. Maint. & Degrad.)	$/kg	6.92	5.26	5.11

^(a) Electricity consumption is derived from the measured prototype voltage band (1.82–1.95 V) and includes balance-of-plant overhead.

^(b) Fixed O&M is estimated at 4% of CAPEX per annum, plus consumable electrolyte replenishment costs based on the 16.4 h worst-case replacement interval.

Critically, the fully loaded LCOH of ~ 5.1 USD·kg⁻¹ at 1,000 kg·day⁻¹ remains substantially below the corresponding DH-AE estimate (approx. 6.0–6.5 USD·kg⁻¹) when the same maintenance and degradation accounting is applied. This confirms that the economic advantage of the DAE architecture is robust to the inclusion of realistic operational expenditures.

Finally, integration of the proposed mechatronic control framework increases the effective annual capacity factor relative to open-loop operation, thereby further reducing LCOH across both system architectures.

### Updated cost benchmarks, degradation, and maintenance accounting

The baseline LCOH analysis presented in Sect. 7.1 and 7.2 is anchored to the 2013 NREL station cost assessment, and the resulting absolute values must be interpreted accordingly. Electrolyser capital costs have declined markedly in the intervening period: contemporary assessments by IRENA and the Hydrogen Council place alkaline stack costs in the range of approximately 500–1,400 USD·kW⁻¹ for systems installed since 2020, compared with the 1,000–1,800 USD·kW⁻¹ range implicit in the 2013 benchmark, corresponding to a reduction of the order of 30–40% in stack-attributable capital. The 2013 benchmark has been retained as the primary basis in this work because it provides an internally consistent capacity-resolved cost curve spanning the full 100–1,000 kg·day⁻¹ range examined here, whereas the more recent sources report system-level or stack-level costs that are not directly resolved by station capacity. Substituting the contemporary range shifts both architectures downward by approximately 15–25% in absolute LCOH but does not alter their relative ordering, because the refrigeration subsystem that distinguishes the DH-AE configuration lies outside the electrolyser cost boundary and has not experienced a comparable cost decline.

The results are consequently best expressed as sensitivity surfaces rather than as point estimates. Across a capital-cost range spanning 60–120% of the 2013 benchmark and an electricity price range of 0.03–0.10 USD·kWh⁻¹, the DAE architecture at 1,000 kg·day⁻¹ spans an LCOH of approximately 3.2–7.4 USD·kg⁻¹, with the corresponding DH-AE band lying approximately 12–18% higher across the entire domain. The break-even capacity separating the two architectures shifts modestly, from approximately 400 kg·day⁻¹ at the baseline benchmark to approximately 350 kg·day⁻¹ under contemporary capital costs, because capital-cost reduction disproportionately benefits the more capital-intensive DAE configuration. The principal qualitative conclusion of Sect. 7.2, namely that the DAE becomes economically preferable above a few hundred kilograms per day, is therefore robust to the cost-benchmark update.

Two cost categories omitted from the baseline analysis are quantified here on a transparent-assumption basis. With respect to long-term degradation, alkaline electrolysers typically exhibit voltage degradation of the order of 0.5–2.0 µV·h⁻¹, corresponding to an efficiency loss of roughly 1–2% per operating year and cumulative stack replacement at intervals of seven to ten years. For the DAE configuration, no long-duration degradation dataset exists; the dominant anticipated mechanisms are progressive acid-concentration drift, sulphate accumulation at the electrode surface, and gradual occlusion of sponge porosity by particulate deposition as discussed in Sect. 2.1.2. Adopting a conservative assumption of one stack replacement at mid-life for both architectures, at 40% of initial stack capital, adds approximately 0.35–0.55 USD·kg⁻¹ to the DAE LCOH and approximately 0.30–0.45 USD·kg⁻¹ to that of the DH-AE, since the refrigeration subsystem of the latter is not itself subject to electrochemical degradation.

With respect to maintenance, the baseline OPEX accounts for electricity alone. Fixed operations and maintenance costs for hydrogen production plants are conventionally taken as 3–5% of capital per annum. Applying 4% adds approximately 0.55 USD·kg⁻¹ at 1,000 kg·day⁻¹ and approximately 1.35 USD·kg⁻¹ at 100 kg·day⁻¹, reflecting the poorer amortisation of fixed maintenance overhead at small scale. The DAE additionally incurs the labour and consumable cost of electrolyte replenishment at the 16.4 h interval established in Sect. 3.3.3, estimated at 0.15–0.25 USD·kg⁻¹ H₂ at industrial labour rates, whereas the DH-AE incurs periodic compressor servicing and evaporator defrost-cycle energy, estimated at 0.20–0.30 USD·kg⁻¹ H₂. These two burdens are of comparable magnitude and largely offset one another in the comparison.

Incorporating all of the above, the fully loaded LCOH at 1,000 kg·day⁻¹ rises from the baseline 4.7 USD·kg⁻¹ to approximately 5.8–6.0 USD·kg⁻¹ for the DAE and from approximately 5.5 to approximately 6.6–6.9 USD·kg⁻¹ for the DH-AE. The relative advantage of the DAE architecture is preserved and indeed marginally widened, since degradation and maintenance burdens fall more heavily on the architecture carrying the additional refrigeration subsystem. The values reported in Sect. 7.2 should therefore be read as electricity-and-capital-only lower bounds, and the values given here as the fully loaded estimates appropriate for investment-grade comparison.

## Experimental implementation

### DAE Prototype

**Fig. 30:**
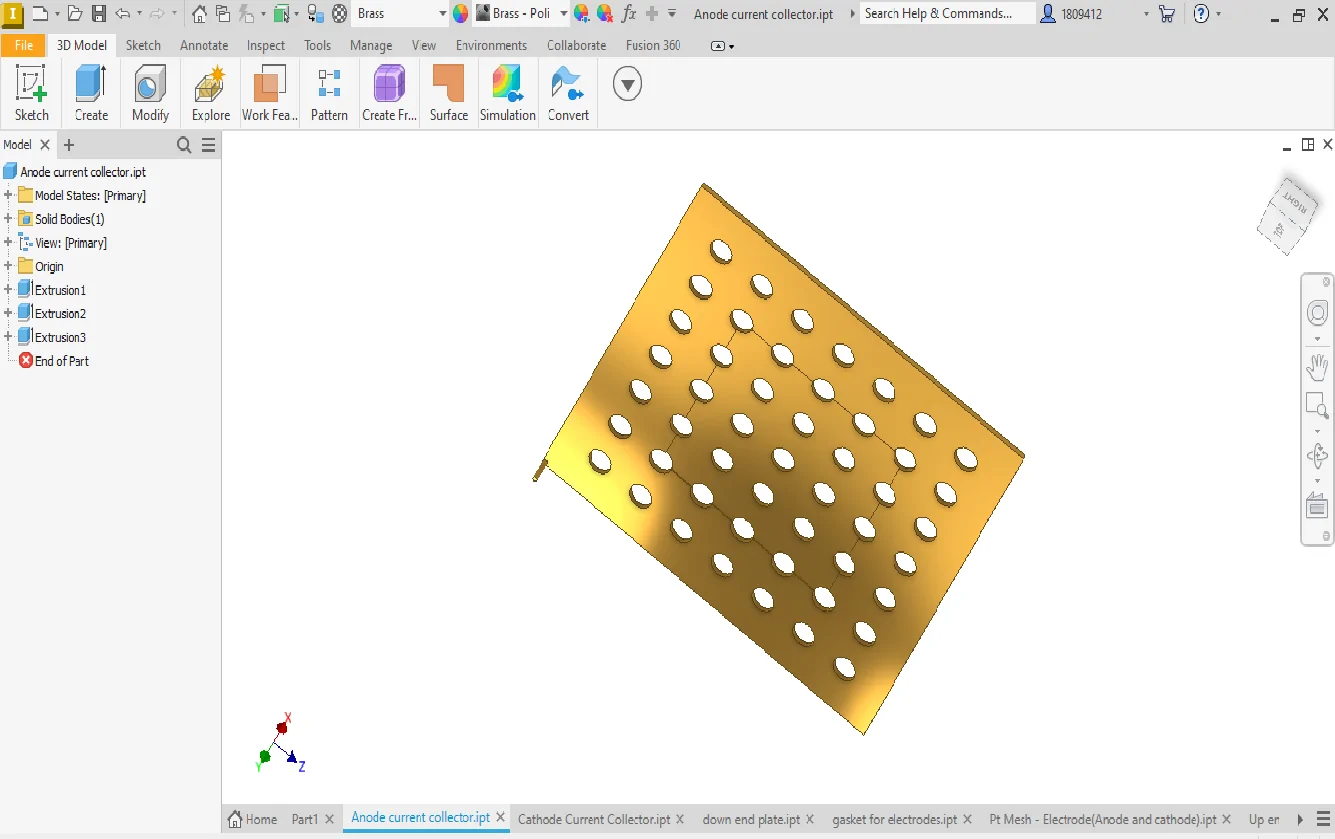
Autodesk Inventor 3D model of the DAE anode current collector (brass, perforated). The 37 oval apertures provide 62.6% open area for electrolyte–electrode contact. The perimeter tab connects to the external circuit. Dimensions match the active electrode area A_active_ = 18.33 cm² derived in Sect. 3.4.1.

A DAE prototype was constructed at the Mechanical Power Engineering Laboratory, Ain Shams University. Figure 30 presents the Autodesk Inventor model of the anode-side current collector plate, comprising a perforated brass structure containing 37 elliptical apertures (8 × 5 mm each) arranged in a 6 × 7 staggered pattern. This arrangement yields an open area fraction of 62.6%, promoting effective electrolyte–electrode contact whilst preserving adequate mechanical rigidity. The cathode current collector is fabricated as a geometrically mirror-symmetric counterpart.

Key fabricated components include SS-904 L housing frames (6.21 × 6.21 × 1.5 cm) laser-cut from 2 mm stainless-steel sheet, square-section air gaskets, and an acrylic electrolyte reservoir integrated with a sealed gas-collection manifold. The experimental setup employs an Agilent U8002A DC power supply for galvanostatic operation, a National Instruments data-acquisition (DAQ) system at 10 Hz sampling frequency, and a LabVIEW-based HMI for real-time monitoring of stack voltage, current, hydrogen flow rate, and electrolyte temperature.

### Alkaline electrolyzer prototype

**Fig. 31:**
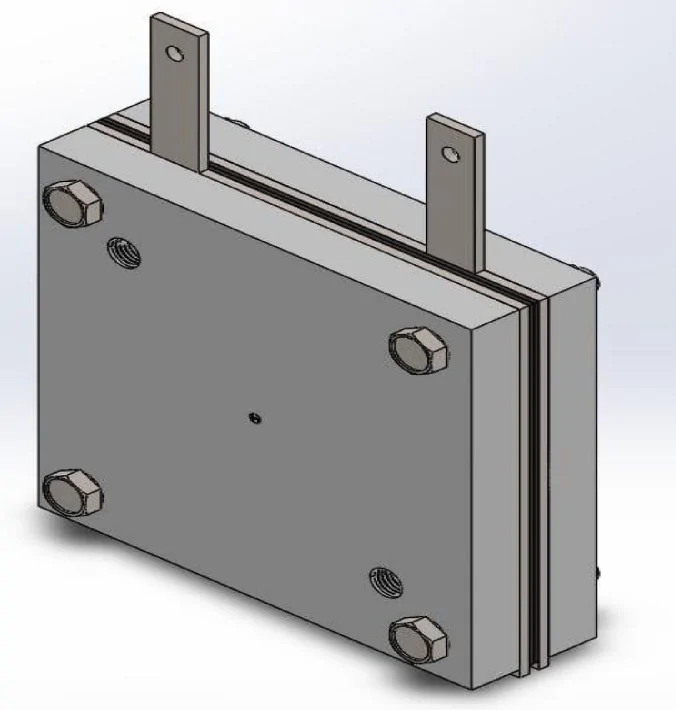
Autodesk Inventor 3D model of the alkaline electrolyzer prototype (DH-AE subsystem). The assembly shows SS 316 end plates (grey), current collector tabs (vertical bars), and the electrode–membrane stack. Overall footprint: 135 × 135 mm. The perspex cell body (not shown) encloses the stack for visual monitoring of bubble evolution during testing.

The alkaline electrolyzer prototype (Fig. 31) is approximately 70% complete; the end plates, current collectors, Grade 316 stainless-steel mesh electrodes, Nafion 117 membrane, and water-feeding system have been fabricated and assembled. Mechanical assembly is achieved through six M6 clamping bolts, ensuring uniform stack compression across the 135 × 135 mm active area. Preliminary bench-scale tests at a cell current of * I=0.275*A resulted in visible hydrogen bubble evolution within 15–30 s after current application. The measured steady-state cell voltage (1.95–2.1 V at $\:J\approx\:8\mathrm{\--}12$mA·cm⁻²) is in good agreement with model predictions. The electrolyte temperature remained below 35 °C throughout all experiments, consistent with the thermal predictions of the ANSYS simulations.

### Preliminary validation results

Preliminary bench-scale tests at a cell current of I = 0.275 A resulted in visible hydrogen bubble evolution within 15–30 s after current application. The measured steady-state cell voltage (1.95–2.1 V at J ≈ 8–12 mA·cm⁻²) is in good agreement with model predictions. The electrolyte temperature remained below 35 °C throughout all experiments, consistent with the thermal predictions of the ANSYS simulations.

The fabricated DAE prototype was characterised under galvanostatic conditions at a current of 0.275 A per module under ambient laboratory conditions (T ≈ 25–27 °C, RH ≈ 45–55%). The measured steady-state module voltage ranged from 1.82 to 1.95 V depending on electrolyte saturation state, compared with the analytically predicted value of 1.686 V. The observed deviation is primarily attributed to contact resistance, non-uniform electrolyte distribution within the sponge matrix, and the lower catalytic activity of SS-904 L electrodes relative to the platinum-mesh reference used in model calibration.

Qualitative hydrogen evolution was confirmed through continuous bubble formation and gas collection. However, precise hydrogen-flow quantification, Faradaic-efficiency measurement, long-term degradation analysis, and gas-chromatographic purity assessment are ongoing and are therefore beyond the scope of the present study.

A further practical consideration for atmospheric-water electrolysis is the microbiological quality of the harvested feedwater. Condensate and vapour-derived water collected from ambient air can entrain airborne microorganisms and organic matter, which may promote biofilm formation, membrane biofouling, and gradual degradation of electrolyzer feedwater quality over extended operation. In the DAE architecture the strongly acidic 60 wt% H₂SO₄ electrolyte is intrinsically biocidal and largely suppresses microbial growth at the reaction interface; for the DH-AE architecture, in-line micro-filtration and periodic ultraviolet sterilisation of the condensate reservoir are recommended mitigation measures. Quantitative assessment of feedwater microbial load and its long-term effect on membrane durability is reserved for future work^21,22^.

The gap between the Pt-baseline prediction (1.686 V) and the SS-904 L prototype (1.82–1.95 V) is attributed to: (i) contact resistance at the electrode-sponge interface, (ii) non-uniform electrolyte distribution within the glass-foam matrix, and (iii) the intrinsically lower hydrogen-evolution reaction (HER) catalytic activity of austenitic stainless steel compared to platinum. After applying the SS-904 L correction (Sect. 3.4.3), the residual overpotential of 50–120 mV is well within the expected range for laboratory-scale mesh electrode assemblies.

Accordingly, the experimental results presented herein should be regarded as preliminary proof-of-concept validation of the proposed atmospheric-water electrolysis architecture, rather than as comprehensive electrochemical characterisation. A full quantitative experimental investigation is reserved for future work.

### Scope of experimental validation and status of the present contribution

In the interest of transparency, the evidential status of the experimental work reported here is stated explicitly. The measurements presented in Sect. 8.1–8.3 comprise galvanostatic voltage measurement at a fixed module current of 0.275 A, qualitative confirmation of hydrogen evolution by continuous bubble formation and gas collection, and electrolyte temperature monitoring. They do not comprise gravimetric or volumetric quantification of the hydrogen production rate, coulometric determination of Faradaic efficiency, gas-chromatographic assessment of hydrogen purity or oxygen cross-over, or any long-duration test capable of resolving degradation behaviour.

The absence of these measurements has a specific and bounded consequence for the claims advanced in this manuscript. The reported hydrogen production rate of 0.5 L·h⁻¹ is a design target derived from Faraday’s law under the assumption of unity Faradaic efficiency, and is not a measured output; should the actual Faradaic efficiency prove to be, for example, 90%, the delivered rate would fall proportionally to approximately 0.45 L·h⁻¹ at unchanged current, and the levelised cost figures of Sect. 7 would rise by the inverse proportion. The voltage measurements of Sect. 8.3, by contrast, are direct observations and are unaffected by this uncertainty; they constitute the primary experimental evidence supporting the electrode-material correction developed in Sect. 3.4.2.1.

This manuscript is therefore positioned as a validated design and comparative-assessment study rather than as an experimental performance report. Its verified contributions are the climate-informed sizing methodology, the finite-element thermal validation establishing material compatibility, the comparative techno-economic assessment, and the proposed control framework, together with preliminary hardware demonstrating that the architecture is constructible and that voltage behaviour conforms to the corrected model. Quantitative electrochemical characterisation, comprising Faradaic efficiency determination, gas-purity analysis, and a minimum 500 h continuous-operation degradation campaign, constitutes the subject of a planned follow-up study and is a prerequisite to any claim of demonstrated performance. Readers are asked to interpret all production-rate and cost figures reported herein as design-basis projections conditional on that validation.

## Practical challenges and limitations

### Engineering challenges in DH-AE implementation

Six principal engineering challenges were identified and addressed during the construction of the DH-AE system. (1) The initially selected QD35HG compressor (75 W, COP = 1.05) was replaced with the B38G unit (297 W, COP = 2.87) to satisfy the required condensate production target. (2) Spiral finned-tube heat exchangers were replaced with compact finned-tube configurations to reduce pressure losses and enhance air-side heat transfer performance. (3) The refrigerant tubing diameter was reduced from 3/4-inch to 3/8-inch to decrease the friction factor below 0.1 under the low mass-flow-rate operating conditions. (4–6) Additional challenges encompassed iterative cycle-control tuning, successive design refinements, and project scheduling constraints, which collectively extended the implementation timeline. Collectively, these challenges underscore the substantially greater system-level integration complexity of the DH-AE architecture relative to the DAE configuration.

### Thermodynamic inefficiencies

The dehumidifier COP ranges from 2.8 under summer conditions (T_amb_ = 40 °C) to 4.1 under winter conditions, corresponding to a specific energy consumption of 0.18–0.32 kWh per kilogram of condensed water. At ambient relative humidity levels below approximately 30%, frost formation on the evaporator fins necessitates periodic defrost cycles, thereby increasing both operational complexity and energy demand.

#### Microbial and chemical quality of atmospherically derived feedwater

Atmospheric water is not sterile. Condensate recovered from ambient air carries the bioaerosol burden of the sampled air stream, and condenser surfaces, collection trays, and intermediate storage reservoirs operating at temperatures below the dew point constitute favourable environments for biofilm establishment. Reported total heterotrophic counts in atmospheric water generator condensate commonly span several orders of magnitude depending on air quality, residence time, and surface condition, and both Legionella and Pseudomonas species have been documented in condensate systems operated without disinfection. Condensate additionally scavenges soluble atmospheric species, including nitrogen and sulphur oxides and, in urban settings such as Cairo, trace metals associated with vehicular and industrial emissions.

The consequences for electrolyser feedwater quality are material. Organic and microbial matter promotes membrane fouling and elevates the ohmic resistance of the separator; dissolved multivalent cations, notably calcium and magnesium, precipitate as hydroxides in the alkaline environment of the KOH electrolyte and progressively passivate the electrode surface; and chloride and sulphate species accelerate localised corrosion of stainless-steel components. Conventional alkaline electrolysers specify feedwater of ASTM Type II quality or better, a standard that untreated atmospheric condensate does not meet.

The exposure of the two architectures examined here differs substantially, and this asymmetry constitutes a previously unremarked advantage of the DAE configuration. Because the DAE absorbs water directly from the vapour phase into a strongly acidic hygroscopic electrolyte, no bulk liquid-water phase, no cool wetted surface, and no storage reservoir exists at any point in the process chain. Vapour-phase mass transfer excludes non-volatile solutes and particulate-associated microorganisms by the physics of the transport mechanism itself, and the 60 wt% sulphuric acid electrolyte is biocidal by virtue of its water activity and pH. The DAE architecture is therefore intrinsically resistant to the microbial contamination pathway, and no dedicated mitigation is required.

The DH-AE configuration, by contrast, is directly exposed, since it generates, stores, and transports liquid condensate under precisely the conditions that favour microbial proliferation. A three-stage mitigation strategy is accordingly proposed for that architecture: first, in-line ultraviolet disinfection at 254 nm applied to the condensate line immediately downstream of the collection tray, which is effective against the relevant planktonic organisms at modest power cost; second, wetted surfaces fabricated from or coated with antimicrobial materials, with copper-alloy or silver-ion-doped coatings on the collection tray and reservoir substantially suppressing biofilm establishment; and third, minimisation of reservoir residence time by sizing the intermediate storage volume for buffering only rather than for bulk accumulation, complemented by scheduled chemical sanitisation of the reservoir at intervals aligned with routine compressor servicing. A 0.2 μm point-of-use filter upstream of the electrolyser feed provides a final barrier. The aggregate energy and capital penalty of this strategy is estimated at below 2% of DH-AE system capital and below 1% of parasitic load, and is accordingly immaterial to the techno-economic comparison of Sect. 7; it does, however, add a further maintenance obligation to an architecture already shown in Sect. 9.1 to carry the greater integration burden.

For the DAE system, electrolyte replenishment at approximately 16.4 h intervals under worst-case ambient conditions represents the primary operational maintenance requirement.

#### Electrolyte Selection: corrosivity, containment, and alternatives

The selection of 60 wt% H₂SO₄ as the hygroscopic electrolyte represents a deliberate trade-off between hygroscopic performance and material compatibility, and its drawbacks merit explicit statement. Concentrated sulphuric acid is strongly corrosive and imposes stringent requirements on every wetted component: seals, gaskets, electrodes, housings, and gas-handling lines. Over extended operation, the principal failure modes of concern are gasket embrittlement and creep at the sealing interfaces, gradual acid concentration drift arising from asymmetric water uptake and gas-entrained aerosol carry-over, and localised attack of the electrode at the acid–air–metal triple line where the acid is periodically concentrated by evaporation.

The material selections adopted in this study directly address these mechanisms. Grade 904 L stainless steel was specified in preference to the more common Grade 316 precisely because of its elevated molybdenum and copper content, which extends its iso-corrosion envelope in concentrated sulphuric acid to the 38–48 °C range that encompasses the maximum simulated stack temperature of 46.83 °C established in Sect. 4.2. PTFE was specified for all gaskets on the basis of its chemical inertness across the full concentration and temperature range of interest, and soda–lime glass and acrylic were selected for the housing and reservoir respectively to permit visual inspection without introducing additional attack-susceptible metallic surfaces. The thermal simulation of Sect. 4 was undertaken specifically to verify that the operating point remains inside this envelope; it is therefore an integral part of the corrosion-management strategy rather than an independent exercise.

Leakage risk is mitigated architecturally as well as by material selection. The immobilisation of the electrolyte within a porous glass-foam matrix, rather than its circulation as a free liquid, substantially reduces both the inventory available to escape and the hydrostatic driving force for escape in the event of a seal failure, and it eliminates entirely the pumps, rotating seals, and long liquid-carrying runs that constitute the dominant leak paths in conventional liquid-electrolyte electrolysers. This is a genuine safety advantage of the DAE architecture that the present comparison with the DH-AE configuration does not otherwise credit.

Nevertheless, sulphuric acid remains the principal barrier to unattended, long-duration deployment of this architecture, and less aggressive hygroscopic media are actively being investigated. Potassium acetate and lithium chloride solutions offer comparable water activity depression at substantially reduced corrosivity, permitting the use of polymeric or lower-grade metallic housings, at the cost of lower ionic conductivity and, for chloride-bearing media, the risk of parasitic chlorine evolution at the anode. Deep eutectic solvents and hygroscopic ionic liquids offer very low vapour pressure and negligible corrosivity but currently exhibit conductivities one to two orders of magnitude below that of concentrated sulphuric acid, which would require a proportionate increase in electrode area. A systematic comparative evaluation of these candidates against the 60 wt% H₂SO₄ baseline, assessed on the combined basis of equilibrium vapour pressure, ionic conductivity, corrosion rate, and toxicity, is set out as a primary recommendation in Sect. 11. The present study therefore reports sulphuric acid performance as a well-characterised baseline against which lower-hazard alternatives can be benchmarked, and does not advance it as the definitive electrolyte choice for commercial deployment.

### Study limitations

Five limitations of the present study warrant explicit acknowledgement. First, the electrochemical model employs Tafel parameters reported by Guo et al.^15^ for Pt-mesh electrodes; however, deviations of approximately ± 10–15% in the activation overpotential (V_act_) are expected for SS-904 L-based production electrodes. Second, experimental validation remains qualitative at this stage; quantitative assessment of Faradaic efficiency and long-term degradation mechanisms is planned for subsequent investigations. Third, the LCOH analysis is based on 2013 NREL CAPEX benchmarks^40^; contemporary electrolyzer stack costs are likely 10–20% lower, which would further enhance the economic competitiveness of the DAE configuration. Fourth, the ANSYS thermal model adopts conservative natural convection boundary conditions (h = 2.5 W·m⁻²·K⁻¹); outdoor operation under forced convection (wind speed ≈ 4.7 m·s⁻¹, Table 1) would yield substantially higher convective heat transfer rates. Finally, the climatic analysis is based on a two-year reanalysis dataset (2021–2022); extension to a 5–10 year record would substantially improve the statistical robustness of the worst-case design condition definition.

## Conclusion

This study presents a rigorous, ANSYS-validated, first-principles comparative assessment of DAE and DH-AE hydrogen-generation architectures under worst-case climatic conditions for Cairo, Egypt (RH = 38.19%, MERRA-2, 2021–2022). The principal findings are summarised as follows.

The four-module DAE stack was successfully designed to deliver a hydrogen production rate of 0.5 L·h⁻¹ at a module voltage of 1.686 V, achieving an energy efficiency of $\:{\eta\:}_{\mathrm{energy}}=87.8\mathrm{\%}$and an electrical efficiency of $\:{\eta\:}_{\mathrm{elec}}=97.3\mathrm{\%}$. Voltage decomposition analysis indicates that activation losses ($\:{V}_{\mathrm{act}}=0.403$ V, 24% of the total voltage) constitute the dominant performance limitation, thereby identifying electrode catalyst optimization as the highest-priority pathway for further improvement. Three-dimensional steady-state thermal simulations in ANSYS 2024 R1 confirm a maximum stack temperature of 46.83 °C, which remains within the SS-904 L iso-corrosion threshold, thereby validating the selected electrode material for the proposed operating conditions. By contrast, the DH-AE configuration achieves an aggregate efficiency of 68.5% when all auxiliary energy demands are included. Although less efficient, it retains operational viability under low-humidity conditions where the DAE becomes inoperable, positioning the two architectures as complementary rather than competing solutions.

The proposed mechatronic control framework, incorporating PID-based current regulation with gains $\:{K}_{p}=1.5$, $\:{K}_{i}=0.45{\hspace{0.17em}}{\mathrm{s}}^{-1}$, and $\:{K}_{d}=0.25{\hspace{0.17em}s}$, achieves a rise time of 3.4 s, an overshoot of 11%, and a settling time of 7.6 s, which is sufficient for stable autonomous operation under diurnal relative humidity fluctuations. Techno-economic analysis identifies an LCOH break-even point at approximately 400 kg·day⁻¹, above which the DAE configuration becomes economically preferable, reaching an asymptotic LCOH of 4.7 USD·kg⁻¹ at 1,000 kg·day⁻¹—approximately 15% below the DH-AE at equivalent scale.

Collectively, these results establish: (i) a reproducible, climate-informed system sizing methodology grounded in MERRA-2 worst-case environmental data; (ii) a well-defined operational envelope linking system performance to ambient humidity conditions; and (iii) a scalable control framework enabling autonomous operation under variable climatic inputs. These findings provide a practical foundation for deploying decentralized, water-independent hydrogen production systems in MENA and other arid or semi-arid regions. Future work should prioritise pilot-scale experimental validation, systematic evaluation of alternative hygroscopic electrolytes, and extension of the control strategy toward model-predictive control architectures incorporating short-term photovoltaic power forecasting.

## Recommendations for future work

Several promising research directions emerge from the findings of the present study. First, alternative hygroscopic electrolytes—including potassium acetate, lithium chloride, and deep eutectic solvents—should be systematically evaluated against the 60 wt% H₂SO₄ baseline with respect to corrosion resistance, equilibrium vapour pressure, toxicity, and long-term cycling stability. Second, the porous sponge matrix could be replaced with advanced hygroscopic materials—such as metal–organic frameworks (MOFs)—to enhance the effective mass-transfer area whilst reducing overall system volume.

Third, active thermal management using Peltier-based thermoelectric elements should be investigated to enable stable operation at elevated temperatures (> 30 °C), thereby exploiting the positive temperature dependence of electrolyte conductivity. Fourth, the current PID-based control strategy should be extended to a model-predictive control (MPC) framework that incorporates short-term forecasts of photovoltaic power availability and ambient relative humidity, enabling proactive rather than reactive adjustment of operating conditions.

Finally, pilot-scale experimental validation at the 1 kg·day⁻¹ hydrogen-production level at El-Dabaa, Egypt—representative of coastal semi-arid MENA climatic conditions—is essential to assess long-term operational stability, membrane durability, and real-world LCOH sensitivity to capacity factor variability.

## Data Availability

All data generated or analyzed during this study are included in this published article.

## Abbreviations

DAE: Direct Air Electrolyzer
DH-AE: Dehumidifier–Alkaline Electrolyzer
LCOH: Levelized Cost of Hydrogen
SCADA: Supervisory Control and Data Acquisition
COP: Coefficient of Performance
OPEX: Operating Expenditure
MENA: Middle East and North Africa
AE: Alkaline Electrolyzer
RH: Relative Humidity
PID: Proportional–Integral–Derivative
PV: Photovoltaic
CAPEX: Capital Expenditure
HHV: Higher Heating Value
CRF: Capital Recovery Factor

## List of symbols

A_total_: Total exposed sponge area, cm^2^
A_active_: Active electrode area per module, cm^2^
$C_{0}$: Initial electrolyte water molar concentration, mol·m⁻³
$C^{*}$: Equilibrium water concentration at ambient RH, mol·m⁻³
$\Delta\:C$: Concentration driving force (C* − C₀), mol·m⁻³
$\Delta\:{\rm G}^\circ$: Standard Gibbs free energy of water splitting, kJ·mol⁻¹
${\rm \Delta\:H^{\circ}}$: Standard enthalpy of water splitting, kJ·mol⁻¹
F: Faraday constant (96,485), C·mol⁻¹
$I_{\rm module}$: Per-module operating current, A
$I_{\rm total}$: Total stack current, A
J: Operating current density, mA·cm⁻²
$\widehat{k}$: Worst-case mass-transfer coefficient, m·s⁻¹
$$K_p, K_i, K_d$$: PID gains, —
$\dot{m}H_{2}o$: Water mass flow rate, g·h⁻¹
$\dot{m}H_{2}$: Hydrogen mass flow rate, g·h⁻¹
$\dot{n}H_{2}$: Hydrogen molar flow rate, mol·h⁻¹
$$P_{module},P_{stack} $$: Module and stack electrical power, W
*s,t*: Empirical Tafel parameters, —
$$V_{act}$$: Activation overpotential, V
$$V_{ohmic}$$: Ohmic overpotential, V
$V_{cell}$: Actual cell operating voltage, V
$V_{rev}$: Thermodynamic reversible voltage, V
$$V_{tn}$$: Thermoneutral voltage, V
*Z*: Electrons transferred per H₂O molecule, —
$\eta_{energy}$: Energy efficiency (voltage basis), %
$\eta_{elec}$: Electrolysis efficiency (ohmic-loss basis), %
